# Foxp3+/CD4+ Cell Ratio in Primary Colorectal Cancer Predicts Opposite Prognoses Following Resection of Synchronous or Metachronous Liver Metastases

**DOI:** 10.1002/cam4.71989

**Published:** 2026-05-29

**Authors:** Andriy Trailin, Esraa Ali, Sergii Pavlov, Wenjing Ye, Lenka Červenková, Ondřej Vyčítal, Filip Ambrozkiewicz, Miroslav Jiřík, Ondřej Daum, Václav Liška, Kari Hemminki

**Affiliations:** ^1^ Laboratory of Translational Cancer Genomics, Biomedical Center, Faculty of Medicine in Pilsen Charles University Pilsen Czech Republic; ^2^ Laboratory of Cancer Treatment and Tissue Regeneration, Biomedical Center, Faculty of Medicine in Pilsen Charles University Pilsen Czech Republic; ^3^ Department of Surgery and Biomedical Center, Faculty of Medicine in Pilsen Charles University Pilsen Czech Republic; ^4^ NTIS, University of West Bohemia Pilsen Czech Republic; ^5^ Sikl's Institute of Pathology, Faculty of Medicine and Teaching Hospital in Pilsen, Charles University Pilsen Czech Republic; ^6^ Bioptická Laboratoř s.r.o. Pilsen Czech Republic; ^7^ Department of Cancer Epidemiology German Cancer Research Center Heidelberg Germany

**Keywords:** colorectal cancer, Foxp3+/CD4+ cells ratio, survival, synchronous and metachronous liver metastases, tumor‐infiltrating lymphocytes

## Abstract

**Background:**

We aimed to assess the abundance, distribution, and prognostic significance of Foxp3+ and CD4+ T‐cells and their ratio in primary colorectal cancer (pCRC) and liver metastases (LM) in patients with synchronous and metachronous disease.

**Methods:**

We performed a retrospective study involving patients who underwent resections of both pCRC and either synchronous (*N* = 55) or metachronous LM (*N* = 44). Following sequential immunohistochemical staining for CD4+ and Foxp3+ T‐cells, whole‐slide scans were analyzed using QuPath software to quantify T‐cells in the tumor center (TC), inner margin (IM), outer margin (OM), and peritumor zone (PT) of both pCRC and LM. T‐cell densities and their ratios were tested as prognostic variables for disease‐free survival (DFS) and time to recurrence (TTR).

**Results:**

We found greater densities of CD4+ cells in OM and PT of LM of synchronous and metachronous patients compared to pCRC. In both groups, densities of Foxp3+ cells were higher in all regions of interest of pCRC compared to LM. CD4+ cells were more abundant than Foxp3+ cells in IM, OM, and PT of LM; densities of Foxp3+ cells were higher in TC and IM of pCRC. Neither CD4+ nor Foxp3+ cells in pCRC were individually predictive of survival, but a higher Foxp3+/CD4+ cells ratio in OM of pCRC in the metachronous group was associated with shorter DFS (hazard ratio (HR): 2.34, 95% confidence interval (CI): 1.14–4.79, *p* = 0.02) and TTR. Conversely, in OM and PT of pCRC in the synchronous group, a higher ratio was associated with longer DFS (HR: 0.53, CI: 0.29–0.98, *p* = 0.04 and HR: 0.46, CI: 0.25–0.86, *p* = 0.02, respectively) and TTR.

**Conclusions:**

The high Foxp3+/CD4+ cells ratio was associated with shorter survival in metachronous and longer survival in synchronous disease, providing novel clinical implications. Foxp3+/CD4+ cells ratio in pCRC may help better stratify CRC patients with synchronous LM after resection of the primary tumor.

## Graphical abstract

The study evaluated the abundance, distribution, and prognostic significance of Foxp3+ and CD4+ T cells, as well as their ratio, in tumor center (TC), inner invasive margine (IM), outer invasive margine (OM) and peritumor zone (PT) of primary colorectal cancer (pCRC) and liver metastases (LM) in patients with synchronous and metachronous disease.

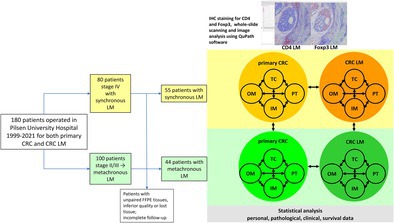

**Abbreviations**
AEC: 3‐amino‐9‐ethylcarbazole
AJCC: American joint committee on cancer
CEA: Carcinoembryonic antigen
CHT: Chemotherapy
CI: Confidence interval
CRC: Colorectal cancer
DFS: Disease‐free survival
FFPE: Formalin‐fixed paraffin‐embedded
FOLFOX: Folinic acid, fluorouracil, and oxaliplatin
Foxp3: Forkhead‐box P3
HR: Hazard ratio
IM: Inner margin
LM: liver metastases
NOS: Not otherwise specified
OM: Outer margin
OS: Overall survival
pCRC: Primary colorectal cancer
PT: Peritumor zone
ROI: Region of interest
TC: Tumor center
Th1: T‐helper 1
Th2: T‐helper 2
TNM: Tumor‐node‐metastasis
Tregs: Regulatory T cells
TTR: Time to recurrence

## 1 Background

Colorectal cancer (CRC) ranks as the third most common cancer worldwide [1] and is the second leading cause of cancer‐related mortality [2]. Distant metastases, a key feature of stage IV CRC, significantly reduce survival rates [3, 4]. About a quarter of CRC patients have already had liver metastases (LM) at the time when the primary CRC (pCRC) is operated (synchronous metastases), while up to a third of patients develop LM later (metachronous metastases) [5, 6]. Surgical resection is the most accepted curative treatment for LM [7], but recurrences occur in up to 70% of patients [8], and the 5‐year survival rate in some reports did not exceed 20% [9, 10]. The majority of research suggests that synchronous metastases exhibit more aggressive traits [5, 6, 11], however, clinical factors do not fully account for differences in survival with respect to the chronicity of LM [12]. Understanding whether there is a biological distinction between synchronous and metachronous metastases could be important for treatment strategies.

The immune system is able to block the development of primary tumor via immune surveillance, as well as hinder the emergence of metastasis via specific immunity triggered by the primary tumor, and conversely, the immune system may also stimulate tumor progression [13, 14, 15]. Tumor‐infiltrating lymphocytes consist of a diverse population of lymphocytes, primarily anti‐tumor CD8+ effector T cells [16, 17], whereas reported prognostic associations of other subsets of T cells have been less conclusive [14, 17].

CD4+ helper T cells play a key role in the adaptive immune response to cancer; these cells comprise several distinct subsets, each with specialized functions and, consequently, markedly different prognostic implications in pCRC [10, 17, 18, 19, 20]. T‐helper 1 (Th1) cells boost cytotoxic T cells and promote anti‐tumor immunity in many cancers including CRC [21], gastric cancer [22] and NSCLC [23]. Other T‐helper subtypes (Th2, Th17, and Tregs) demonstrated variable associations depending on cancer type [21], however, in CRC they were more frequently associated with inferior survival [10, 17].

Tregs, which have recently gained considerable attention, are primarily CD4+ T cells that are characterized by the expression of the IL‐2 receptor alpha chain and the transcription factor forkhead‐box P3 (Foxp3) [14, 24]. Foxp3 is essential for the development of these cells, and it is currently the most commonly used marker for analyzing Tregs by immunohistochemistry [14, 24, 25]. Although tumor‐infiltrating Tregs are associated with a dismal prognosis in many cancers including lung, breast, ovarian, and hepatocellular as reviewed in [23, 24], discrepant data have been reported for CRC, from pro‐tumor to anti‐tumor [10, 19, 24, 26, 27], which is probably related to the heterogeneity of Tregs.

Published results on CD4+ and Foxp3+ T cells in CRC LM are scant [10, 28, 29, 30]. Only a few studies compared the distribution and prognostic performance of Foxp3+ T cells [31, 32, 33] between pCRC and LM, with a focus on the synchronous setting [34]. Discrepant prognostic associations of the Foxp3+ to CD4+ cell ratio were reported with respect to cancer type: no associations in esophageal squamous carcinoma [35], favorable associations of a high ratio in breast cancer [36] and unfavorable in bladder cancer [37]. Katz et al. demonstrated the greater prognostic implication of the Foxp3+ to CD4+ ratio in mixed synchronous and metachronous CRC LM compared to those cells alone [30].

Despite substantial scientific research, several key questions concerning Foxp3+ and CD4+ T cells in CRC remain unanswered. These include: [1] whether tumor‐infiltrating T cells underlie the differences in clinical course and prognosis between patients with synchronous versus metachronous CRC; [2] whether the prognostic relevance of T cells in pCRC differs from that in LM; [3] whether prognosis in CRC is more strongly associated with CD4+ and Foxp3+ T cells individually or with their ratio; and [4] whether the spatial distribution of T cells across distinct regions of interest (ROIs) in pCRC and LM is linked to their prognostic significance.

In this paper we aimed to assess and compare the abundance, regional distribution, and prognostic significance of Foxp3+ and CD4+ T cells alone and their ratio in pCRC and LM and in patients with synchronous and metachronous disease.

## 2 Methods

### 2.1 Patients

Detailed description of the cohort and methods is provided in our earlier paper [38]. In brief, all patients who underwent a resection of pCRC followed by liver resection for the first recurrence of CRC at Pilsen University Hospital from 1999 till 2021 were retrospectively identified. Patients diagnosed with LM at the time of pCRC diagnosis were classified as stage IV, synchronous. In contrast, stage I‐III patients, who developed LM after resection of the primary tumor, were categorized as the metachronous [29].

Inclusion criteria were: only the first LM, curative‐intent resection of both pCRC and LM, availability of complete clinical and survival data, and availability of high‐quality formalin‐fixed paraffin‐embedded (FFPE) tissue samples of pCRC and LM in the pathology archive of Sikl's Institute of Pathology. The final cohort consisted of 55 stage IV patients and 44 stage I‐III patients. Figure S1 shows the flowchart of the study.

Relevant information was obtained from the patients' archives and medical records to document basic demographic, pathological, and clinical details (Table 1). Tumor's clinical stage was determined according to the eighth edition of the American Joint Commission on Cancer [39]. This retrospective study was performed in compliance with the ethical standards outlined in the Declaration of Helsinki (2013 version) and was approved by the Ethics Committee of the Faculty of Medicine and University Hospital in Pilsen (300/2020, 17 June 2020).

**TABLE 1:** Clinical backgrounds of enrolled patients and histopathological features of primary and metastatic tumors.

Parameter		Synchronous group, *N* = 55	Metachronous group, *N* = 44	*p*
Age at the diagnosis (years), median (min‐max)		62 (29‐78)	64 (46‐73)	0.31
Gender	male	34 (61.8%)	29 (65.9%)	0.67
	female	21 (38.2%)	15 (34.1%)	
Primary tumor				
Location	Right colon	12 (21.8%)	8 (18.2%)	0.65
	Left colon	43 (78.2%)	36 (81.8%)	
Size (cm), median (min‐max)		4.3 (1.0‐7.5)	3.5 (0.8‐8.3)	0.06
Pathologic T stage	T1 T2 T3 T4	0 (0.0%) 1 (1.8%) 48 (87.3%) 6 (10.9%)	1 (2.3%) 6 (13.6%) 35 (79.5%) 2 (4.5%)	0.25
Histological type	NOS Mucinous Other	51 (92.7%) 2 (3.6%) 2 (3.6%)	39 (88.6%) 4 (9.1%) 1 (2.3%)	
Pathologic N stage	N0	13 (23.6%)	13 (29.5%)	0.51
	N1	22 (40.0%)	16 (36.4%)	
	N2	20 (36.4%)	15 (34.1%)	
AJCC 8th staging	Stage I Stage II Stage III Stage IV	55 (100%)	1 (2.3%) 12 (27.3%) 31 (70.4%)	
Grade	1 2 3	12 (21.8%) 37 (67.3%) 6 (10.9%)	14 (31.8%) 26 (59.1%) 4 (9.1%)	0.77
*KRAS* status	mutated	20 (36.4%)	11 (25.0%)	0.26
	WT	27 (49.1%)	25 (56.8%)	
	not tested	8 (14.5%)	8 (18.2%)	
*BRAF* status	mutated	0 (0.0%)	1 (2.3%)	0.27
	WT	44 (80.0%)	35 (79.5%)	
	not tested	11 (20.0%)	8 (18.2%)	
CEA ng/ml		6.8 (1.0‐945.0)	8.9 (0.8‐1649.0)	0.90

Parameter		Synchronous, *N* = 55	Metachronous, *N* = 44	*p*
CEA	< 5ng/mL	18 (46.2%)	13 (46.4%)	
	> 5ng/mL	21 (53.8%)	15 (53.6%)	0.98
Number of examined lymph nodes, median (min‐max)		13 (1‐34)	11 (0‐43)	0.38
Lymph node ratio, median (25‐75perc)		0.22 (0‐0.57)	0.13 (0‐0.32)	0.14
Liver metastases				
Number, median (min‐max)		2 (1‐24)	1 (1‐7)	0.08
Size (cm), median (min‐max)		2.0 (0.4‐26.0)	2.7 (0.6‐7.1)	**0.01**
Grade	1	21 (39.6%)	18 (42.9%)	0.75
	2	32 (60.4%)	24 (57.1%)	
Metastasis resection R status	R0	40 (76.9%)	29 (69.0%)	0.39
	R1	12 (23.1%)	13 (31.0%)	
Chemotherapy ± biological regimens	CHT alone	28 (50.9%)	30 (68.2%)	0.07
	CHT + anti‐VEGF	11 (20.0%)	4 (9.1%)	
	CHT + anti‐EGFR	9 (16.4%)	5 (11.4%)	
	no treatment	4 (7.3%)	2 (4.5%)	
	not known	3 (5.4%)	3 (6.8%)	
Chemotherapy regimens	FOLFOX	31 (56.4%)	16 (36.4%)	**0.048**
	dle Gramont	7 (12.7%)	9 (20.5%)	
	FOLFIRI	4 (7.3%)	2 (4.5%)	
	others	6 (10.9%)	12 (27.3%)	
Chemotherapy ± biological therapy timing	Before liver surgery	37 (67.3%)	11 (25.0%)	**0.0001**
	After liver surgery	12 (21.8%)	32 (72.7%)	
	No	6 (10.9%)	1 (2.3%)	

*Note:* Bold values indicate statistical significance at the *p* < 0.05 level.

Abbreviations: AJCC: the American joint committee on cancer; CEA: the carcinoembryonic antigen; CHT: chemotherapy; NOS: not otherwise specified.

### 2.2 Pathology and Immunohistology

FFPE tissue blocks of pCRC and LM from each patient were cut into 4‐μm sections. For pCRC, the block with the largest surface of the tumor, deepest invasion and the least necroses was selected. In case of multiple LMs, we selected the one with the least regressive changes, which was also the least infiltrated by immune cells [40]. One or two tissue sections from one FFPE block were mounted onto BOND Plus Microscope Slides (Cat# 00270, Leica Biosystems Newcastle Ltd., Newcastle, UK). Sequential immunohistochemical detection of CD4+ and Foxp3+ T cells was performed using a fully automated BOND RX^m^ stainer. Ready‐to‐use monoclonal primary antibodies for CD4 (clone 4B12) and Foxp3 (clone 236A/E7), all from Leica Biosystems (Newcastle Ltd., United Kingdom), were used (see detailed procedure in Supporting Information and methods). A pilot study aimed at establishing the protocol and validating the antibody preceded the main study. Slides were first stained for CD4, then the binding of primary antibodies with their targets was visualized using horseradish peroxidase (HRP)‐linker antibody conjugate system (Bond Polymer Refine Detection) and alcohol‐soluble chromogen 3‐amino‐9‐ethylcarbazole (AEC) (Biolegend). Sections were counterstained with hematoxylin, mounted into glycergel (C0563, Dako), and scanned. After scanning, slides were decoverslipped in the warm dH_2_O, and AEC was washed out in ethanol solutions of increasing concentration until complete destaining, which was controlled microscopically. Sections were rehydrated and antibodies to CD4 were stripped by prolonged heat‐induced epitope retrieval (Supporting Information and methods). Then, sections were stained for Foxp3 followed by the HRP‐linker antibody conjugate system with diaminobenzidin as a chromogen (Bond Polymer Refine Detection).

Sections were counterstained with Mayer's hematoxylin and embedded into Micromount mounting medium (Leica Biosystems Newcastle Ltd., United Kingdom). Appropriate positive (tonsils) and negative tissue control samples were used throughout.

### 2.3 Image Analysis

Whole‐slide scans were obtained using Olympus VS200 scanner (Olympus, Shinjuku, Japan) at objective 20× and saved in .vsi file format. One section of pCRC and LM per patient was analyzed. Using the “wand” tool of open‐source software QuPath v.0.3.2 [41] a border separating the malignant cell nests and adjacent non‐tumor tissue was drawn in all samples by an expert image analyst and human pathologist (AT), and the accuracy of annotations was validated then by the expert pathologist (OD). Subsequently, image analysts (EA, SP and WY) used a script (https://github.com/sergii01‐cuni/script_zones) integrated into QuPath to automatically extend inner margin (IM) and outer margin (OM) as 500 μm‐wide regions on each side of the border, as recommended by [42]. Tumor center (TC) represents the remaining tumor area. The peritumor zone (PT) was defined as the 500 μm‐wide regions adjacent to the OM (Figure S1B).

Before analysis, all lumina, non‐tumor tissue, necroses, and artifacts were excluded as recommended [43] and described in detail earlier [38]. Density of CD4+ and Foxp3+ cells was estimated as the number of immunopositive cell profiles divided by the total area of ROI. To reduce skewness in the distribution, the raw data were converted into percentile values and classified into Low (below the 25th percentile) versus High (25th–100th percentile) to define a biologically relevant low‐density subgroup [44]. This approach enhances contrast with the remaining cohort compared with median‐based dichotomization, improving sensitivity for clinically meaningful associations. Binary stratification also avoids reduced statistical power and interpretative complexity associated with multi‐tier classification, with comparable results reported previously [44].

### 2.4 Follow‐Up

Patients were monitored until December 2023, with a median follow‐up time after metastasectomy of 84 months (95% confidence interval (CI): 5–163 months) in the synchronous group and 61 months (95% CI: 54–68 months) in the metachronous group, as described earlier [38].

### 2.5 Outcomes

Survival was compared first since date of colon surgery in synchronous and metachronous groups. Patients from the metachronous group showed significantly longer DFS, TTR, and OS after pCRC surgery compared to the synchronous group (Figure S2). To avoid the guarantee‐time bias and compare outcomes between synchronous and metachronous groups, the day of liver surgery was chosen then as the reference date [45]. The primary endpoint was disease‐free survival (DFS) that was considered as the time from resection of LM to the date of diagnosis of recurrence or death from any cause. Secondary outcomes were time to recurrence (TTR) and overall survival (OS).

### 2.6 Statistical Methods

We compared cell densities (1) between different ROIs within pCRC or LM, (2) between corresponding ROIs of pCRC and LM, (3) between corresponding ROIs of pCRC or LM of synchronous and metachronous groups (Figure S1C). We evaluated the prognostic impact of factors related to the patient, primary, and metastatic tumors for DFS, TTR, and OS. We first assessed the prognostic impact of individual immune cell types in each ROI of pCRC and LM. Next, for each location and ROI, we calculated Foxp3+/CD4+ cells ratio and performed dichotomization into the above‐median and below‐median groups. Finally, for each cell type, we calculated ratios of cell densities between IM and OM and performed dichotomization into the above‐median and below‐median groups to characterize the efficiency of penetration of immune cells into the tumor.

Non‐normally distributed continuous data are expressed as median (min‐max), and compared either by Mann–Whitney U‐test or by Friedman ANOVA, followed by Wilcoxon matched pairs test. Proportions are expressed as raw data (percentages). The associations between pairs of ordinal or quantitative variables were assessed using Spearman correlation due to nonparametric distribution of most of the variables. DFS, TTR and OS were estimated using the Kaplan–Meier method and compared between groups with the log‐rank test. Cox regression analysis was performed to determine the prognostic value of individual predictors for TTR, DFS and OS, with Low and below‐median as reference categories. GraphPad Prism 9.0 (GraphPad Software LLC) and R software environment (ver. 4.2.1) were used for the statistical analyses. Survival analysis was performed in the R environment with the Finalfit package [46]. Kaplan–Meier analysis was performed with the survival package and plots were generated with the survminer package [47, 48]. A 2‐sided *p* < 0.05 was considered statistically significant.

## 3 Results

### 3.1 Characteristics of CRC Patients

The demographic, clinical and pathological characteristics of the patients are shown in Table 1. DFS, TTR, and OS after LM surgery were comparable between groups (see Supplementary Figure S3).

Patients with synchronous and metachronous LM differed only in median size of LM, which was greater in the metachronous group and in proportion of patients who received FOLFOX chemotherapy, which was lower in the metachronous group (Table 1).

### 3.2 Distribution of T Cells in pCRC and LM at the Group Level

**FIGURE 1:**
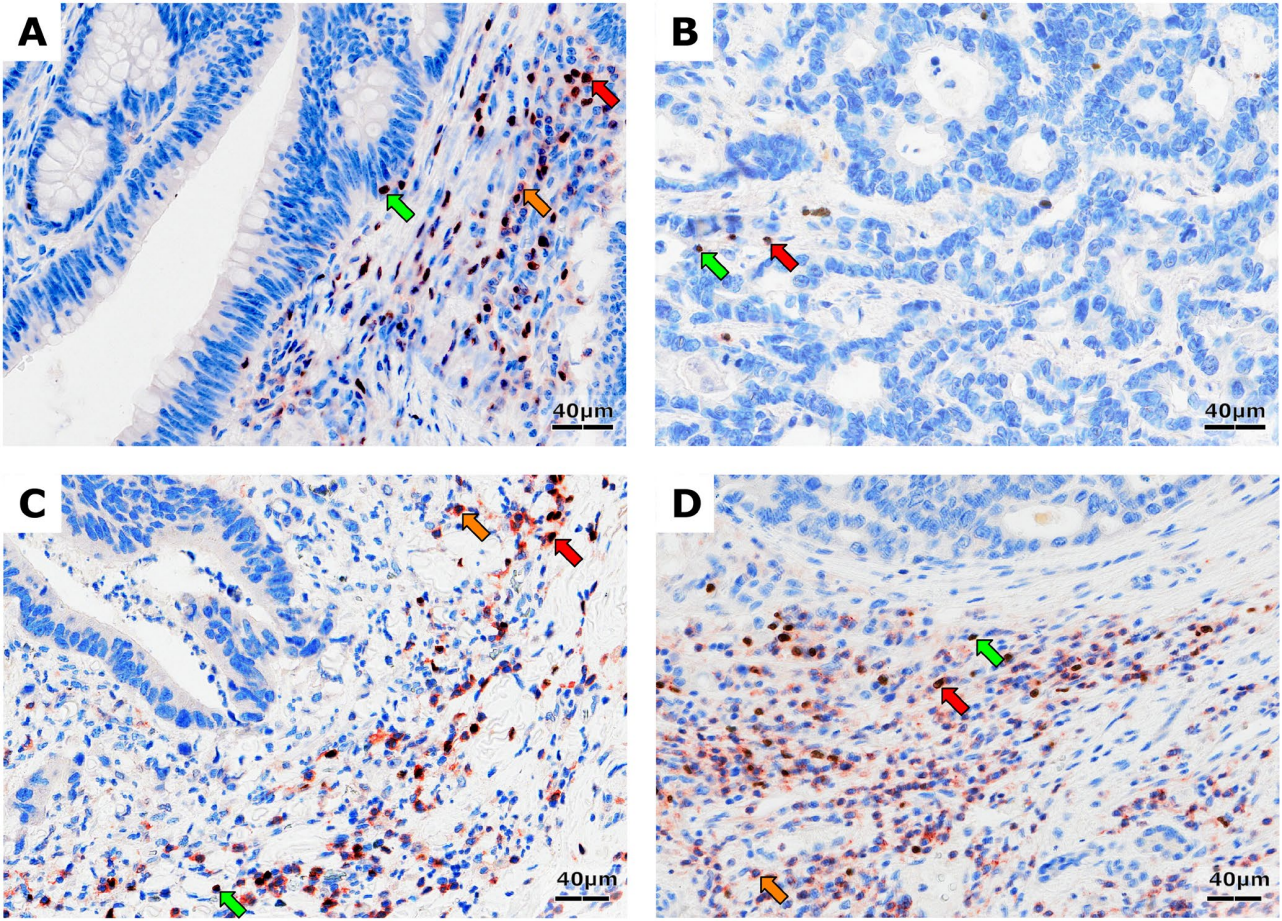
Representative immunostaining of CD4+ and Foxp3+ cells in the tumor center (A) and invasive margin (C) of primary colorectal cancer and in the tumor center (B) and invasive margin (D) of liver metastases of colorectal cancer. Single‐positive Foxp3 cells (brown nucleus, DAB chromogen, green arrow) and CD4 cells (red cytoplasm, AEC chromogen, orange arrow) and double‐positive cells (red arrow). Objective 20×.

**FIGURE 2:**
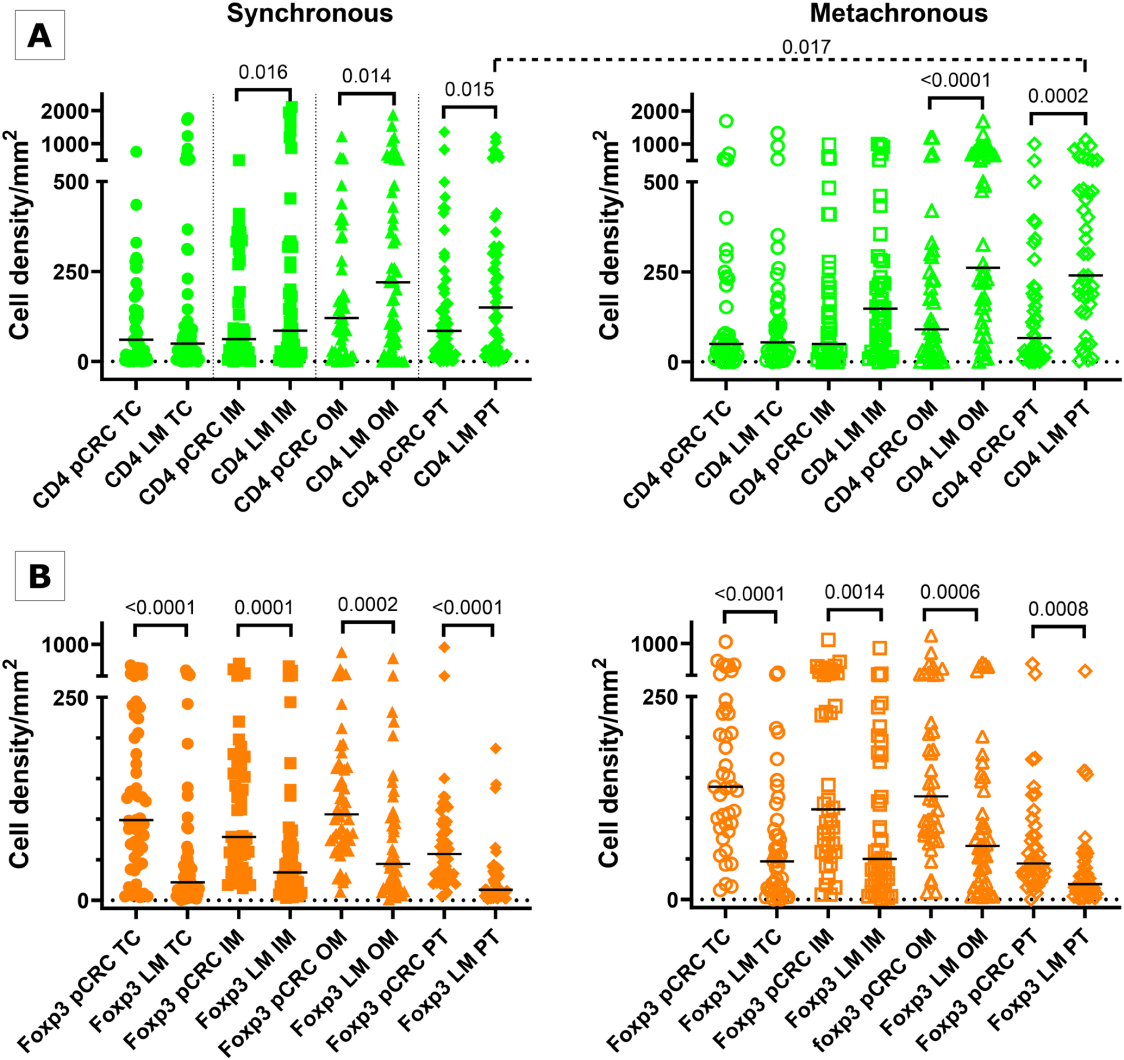
Statistics depicting the spatial distribution of CD4+ (A) and Foxp3+ (B) tumor infiltrating lymphocytes per mm^2^ of the section in the TC, IM, OM and PT of pCRC and LM in patients with synchronous (left panel, filled symbols) and metachronous disease (right panel, empty symbols). Black lines: Medians. IM, Inner invasive margin; LM, Liver metastases; OM, Outer invasive margin; pCRC, Primary tumor; PT, Peritumor zone; TC, Tumor center.

Foxp3+ and CD4+ cells were principally found in the stromal compartment of the tumor. In OM and PT the majority of CD4+ T cells were located in organized aggregates of lymphocytes (Figure S4). We observed co‐localized expression of Foxp3 and CD4 in many cells; however, single‐positive cells were also present in each ROI (Figure 1). We compared densities of T cells between pCRC and LM in the synchronous and metachronous groups (Figure 2). CD4+ cell densities in both groups were greater in LM compared to pCRC in OM and PT, and additionally in synchronous IM (Figure 2A). Cell densities were the highest in LM OM and PT. Densities of Foxp3+ cells were higher in pCRC compared to LM, and this was observed in the synchronous and metachronous groups and in all ROIs (Figure 2B). The only difference in the density of T cells between the synchronous and metachronous groups was for CD4+ T cells, with higher densities in PT of metachronous LM compared to that ROI in the synchronous group (dashed significance bar on top of Figure 2A).

Comparison of cell densities between CD4+ and Foxp3+ showed significant excess of the former in LM IM, OM and PT (*p* < 0.001); conversely, densities of Foxp3+ cells were higher in pCRC TC and IM (*p* < 0.05).

### 3.3 Correlation Between T Cells in pCRC and LM

Results of correlation analysis of T cell densities between ROIs of pCRC and LM are shown in Table S1.

CD4+ T cell densities correlated between the majority of ROIs in the synchronous group, whereas in the metachronous group concordant correlation was observed for OM and PT in pCRC and for OM in LM. Foxp3+ T cells densities correlated between the majority of ROIs in the metachronous group, whereas in the synchronous group no correlations were found for PT. Significant correlations between CD4+ and Foxp3+ T cells were observed in all ROIs of metachronous pCRC and in most ROIs of synchronous pCRC (Table S2). Significant correlations between CD4+ and Foxp3+ T cells were also observed in all but one ROI of LM; some of these correlations were at or over 0.7 in concordant ROIs, such as TC and IM in synchronous LM and IM and OM in metachronous LM (Table S2).

### 3.4 Association Between Cell Densities and Survival

Initially, we analyzed the associations between T cell densities in individual ROIs of pCRC and LM with outcomes and found no associations with DFS and TTR (Tables S3 and S4) in neither the synchronous nor the metachronous groups. As for OS, only high densities of CD4+ T cells in LM (TC) of the metachronous group were associated with a lower risk of death: HR = 0.27 (95% CI: 0.12–0.63), *p* = 0.002 (Table S5).

**FIGURE 3:**
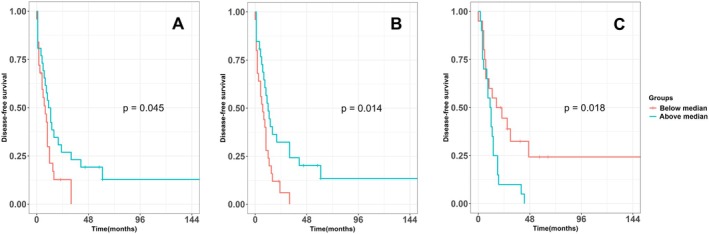
Kaplan–Meier analysis for DFS according to above median vs. below median Foxp3 to CD4 ratio of T cell density per individual ROI of pCRC in CRC patients with synchronous and metachronous metastases. *p* values according to log‐rank test. (A) pCRC (OM) in the synchronous group, (B) pCRC (PT) in the synchronous group, (C) pCRC (OM) in the metachronous group. LM, Liver metastases; OM, Outer invasive margin; PT, Peritumor zone; TC, Tumor center.

Above median ratio Foxp3+/CD4+ cells in pCRC (OM and PT) in the synchronous group was associated with a higher DFS in Cox regression (Table 2) and longer DFS in Kaplan–Meier analysis (Figure 3A,B).

**TABLE 2:** Hazard ratios for DFS between above median vs. below median Foxp3 to CD4 ratio of T cell density per individual ROI of pCRC in CRC patients with synchronous and metachronous metastases.

Cell type and location	Synchronous, *N* = 55			Metachronous, *N* = 44		
		*N* (%)	HR (95% CI), *p*		*N* (%)	HR (95% CI), *p*
TC	Above median	26 (50.0)	1.34 (0.74‐2.42), *p* = 0.33	Above median	20 (50.0)	1.85 (0.92‐3.71), *p* = 0.08
IM	Above median	26 (51.0)	0.62 (0.34‐1.14), *p* = 0.12	Above median	20 (50.0)	1.09 (0.56‐2.15), *p* = 0.80
OM	Above median	26 (51.0)	**0.53 (0.29‐0.98), *p* = 0.04**	Above median	20 (50.0)	**2.34 (1.14‐4.79), *p* = 0.02**
PT	Above median	26 (51.0)	**0.46 (0.25‐0.86), *p* = 0.02**	Above median	22 (55.0)	1.82 (0.89‐3.70), *p* = 0.10

*Note:* Raw densities of Foxp3+ and CD4+ T cells per area of ROI (mm^2^) were used to calculate FoxP3/CD4 ratio and then categorize patients into above and below median groups. Hazard ratios show the relative risk compared with 1.00 for below median group. Bold values indicate statistical significance at the *p* < 0.05 level.

Abbreviations: CI, confidence interval; CRC, colorectal cancer; DFS, disease‐free survival; HR, hazard ratio; IM, inner margin; OM, outer margin; pCRC, primary colorectal cancer; PT, peritumor zone; ROI, region of interest; TC, tumor center.

Contrary to this, in the metachronous group above median ratio Foxp3+/CD4+ cells in pCRC (OM) was associated with a lower DFS in Cox regression (Table 2) and shorter DFS in Kaplan–Meier analysis (Figure 3C). Concordant associations were observed for TTR (Table S6), but not for OS (Table S7). No associations were found for Foxp3+/CD4+ cells ratio in LM (Tables S6–S8). Above median IM to OM ratio of Foxp3+ cells in LM and in the metachronous group only was associated with a higher DFS in Cox regression (Table S9) and longer DFS in Kaplan–Meier analysis (Figure S5). Concordant associations were observed for TTR, but not for OS (Table S9).

### 3.5 Prognostic Associations of Clinical and Pathological Variables and Their Associations With Immune Cells

Among clinical and pathology variables only older age was associated with longer TTR and only in the metachronous group (Table S10). In addition, patients in the metachronous group receiving non‐FOLFOX regimen of chemotherapy demonstrated shorter OS: 2.79 (95% CI: 1.04–7.51), *p* = 0.043. Most of prognostic associations remained significant in multivariate analysis in metachronous group after adjustment for the effects of age and chemotherapy regimen (Table S11). Timing of chemotherapy did not have any prognostic associations (Table S12). Associations of T cell densities with clinical and pathological variables are shown in Table S13. Patients from metachronous group, who received chemotherapy before liver surgery, had smaller densities of CD4+ and Foxp3+ T cells in LM OM (Table S14). Foxp3+/CD4+ cells ratio did not differ with respect to timing (Table S14) or regimen of chemotherapy (Table S15).

## 4 Discussion

The scope of the present study was evaluating fine regional distribution and prognostic value of Foxp3+ and CD4+ T‐cells in pCRC and LM in CRC patients with synchronous and metachronous disease. Contrary to Foxp3+ or CD4+ cells alone, we identified prognostic associations of high Foxp3+/CD4+ cells ratio in pCRC, which were anti‐tumor in the synchronous group and pro‐tumor in the metachronous group.

### 4.1 Prognostic Significance of Foxp3+ to CD4+ T Cells Ratio in pCRC Is Anti‐Tumor in Synchronous Group and Pro‐Tumor in Metachronous Group

Higher Foxp3+/CD4+ cells ratio in OM of pCRC in the metachronous group was associated with shorter DFS and TTR, which is in line with putative anti‐tumor effects of CD4+ T cells and pro‐tumor effects of Foxp3+ cells, generally associated with CRC progression and metastasis [10, 14, 17, 27, 49]. Antitumor effect of CD4+ T cells can be elicited in case of their polarization towards Th1 cells that boost effector functions of cytotoxic T cells and promote anti‐tumor immunity [12, 16, 17]. Observed pro‐tumor effects of Tregs might be chiefly associated with predominance of immunosuppressive Tregs, and it is supported by one earlier report on rectal cancer [49]. Tregs in pCRC can induce cell‐cycle arrest or apoptosis of T‐effector cells, NK cells, and dendritic cells [14, 25, 26]. Tregs can also compete for T cell growth factors such as IL‐2, and in direct contact inhibit effector immune cells via CTLA‐4 [13, 14]. Altogether, Tregs may promote metastasis by limiting immunosurveillance in the primary tumor (reviewed in [13]). In addition, Tregs can support tumor cells by releasing growth factors and interacting with stromal cells [25]. In a spontaneous metastasis model of breast cancer, the suppression of lung metastasis was accompanied by lower numbers of CD4 + CD25+ cells in the primary tumors, which suggests that Treg recruitment to the primary tumor is necessary to enable metastasis [50]. Our data on the metachronous group support the notion that inhibition of Tregs may prevent metastasis [13]; indeed, anti‐CD25 therapeutic antibodies have been shown to synergize with anti‐PD1 treatment [31].

Contrarywise, a higher Foxp3+/CD4+ cells ratio in OM and PT of pCRC in the synchronous group was associated with longer DFS and TTR, which suggests the anti‐tumor effects of Foxp3+ cells along with pro‐tumor effects of CD4+ cells. Anti‐tumor effects of Foxp3+ cells imply polarization of Tregs into their proinflammatory states, such as fragile Tregs, unstable Tregs, and Th1‐like Treg, which produce IFN‐γ and IL‐17, and can contribute to the destruction of tumor cells [25]. Additionally, CD4+ T helpers can be polarized towards Th2 and Th17 cells with immunosuppressive effects [12, 16, 17].

Discordant findings in synchronous and metachronous groups can be explained through significant plasticity of Tregs and T‐helper cells in different microenvironments [10, 14, 15, 25, 28] enabling existence of phenotypically similar subsets with different functions, from immunosuppressive pro‐tumor to proinflammatory anti‐tumor. The majority of cancers are infiltrated primarily by highly immunosuppressive Tregs, whereas in certain cancers, including CRC, both immunosuppressive and proinflammatory Tregs can be present [10, 51, 52]. It is plausible, therefore, to hypothesize that the opposite prognostic effects of T‐helpers and Tregs in our study may relate to the functional heterogeneity of both populations with respect to tumor stage and chronicity status of LM. We hypothesize that the state of polarization and activity of T cells in synchronous pCRC can be modified by the presence of synchronous LM, or this is an effect of a larger tumor mass, which is supported to some extent in the literature [13, 53, 54]. Earlier on the same cohort we observed the anti‐tumor associations of CD8+ T cells only in synchronous LM [38]. Besides CD4+ and Foxp3+ T cells, the immune microenvironment of synchronous and metachronous CRC differs with respect to other immune cell subsets, such as cytotoxic and memory T cells, which shape subsequent outcomes differently, as shown by us [38] and other authors [55]. Different prognosis conferred by Tregs in synchronous and metachronous pCRC is in line with reported prognostic associations of local Tregs in different clinical stages of gastric cancer [56]. As for pCRC, both worse and good prognostic associations of local Tregs have been reported [10, 14]. Heterogeneity of Tregs highlights the importance of multiplex analysis for accurate assessment of Tregs and other immune cells in tumors in relation to patient outcome. Katz et al. 2013 on the cohort with mixed synchronous and metachronous LM revealed that high Foxp3+/CD4+ cells ratio in LM predicted shorter OS, whereas Foxp3+ cells alone were not predictive [30]. These observations suggest that the composition of the immune infiltrate, facilitating interactions between different immune cell subsets, is more important than its overall volume [57]. The nature of the immune response is determined at the stage of antigen presentation, with the balance between T effector cells and T regulatory cells playing a crucial role in shaping the final outcome [58].

With respect to the clinical translation of our findings, we consider them particularly relevant for patients with synchronous disease, who exhibited worse prognosis in our study as well as in previous reports [5, 6, 7]. Foxp3+/CD4+ cell ratio assessment in pCRC by ubiquitously available immunohistochemistry may serve as an additional tool to better stratify CRC patients with synchronous LM. One more additional value of Foxp3+/CD4+ cell ratio could be better stratification of patients within particular consensus molecular score in order to improve prognostic stratification, predict response to immunotherapy or tailor immunomodulation [59].

The last, but not least, observation was that only cells in OM and PT showed prognostic associations, which emphasize the importance of those ROIs and might require adjustment of current guidelines for assessment of tumor‐infiltrating lymphocytes [42].

### 4.2 CD4+ Cells Predominate in the Tumor Exterior of LM and Foxp3+ Cells Predominate in the Tumor Interior of pCRC

We highlighted a significant excess of CD4+ T cells in the OM and PT of both the synchronous and metachronous LM, which can be attributed to the presence of organized lymphoid structures, which are enriched in CD4+ T cells, in these ROI. Conversely, densities of Foxp3+ cells were higher in TC and IM of pCRC, which correspond to earlier reports on Foxp3+ [28, 34] and CD4+ cells [60, 61] in mixed or synchronous LM. Multiple factors contribute to the increased frequency of Treg cells in tumors over normal tissues [24]: attraction by chemokines produced in the tumor microenvironment, conversion from CD4+ T cells, increased proliferation and stability of Foxp3 expression.

### 4.3 CD4+ Cells in the Microenvironment of Metachronous LM Are Independent From pCRC

We observed more correlations between CD4+ cells in pCRC and synchronous than metachronous LM that points to relative independence of the immune microenvironment of pCRC and metachronous LM. Our findings indicate that comparative assessment of the primary tumor and LM should take into account chronicity status. Such results also do not support the concept [15, 62] of similarity of immune status between primary and metastatic tumor and that the assessment of the local immunity of the primary tumor may substitute the evaluation of the metastatic lesion.

### 4.4 Comparisons With Relevant Literature

Several earlier papers reported results discordant to ours regarding the distribution of Foxp3+ and CD4+ cells in pCRC and LM and between different ROIs [28, 31, 63] and their prognostic associations [18, 19, 20, 64]. Such discrepancies can be attributed to semiquantitative assessment in selected microscopic fields of view of arbitrarily defined ROI (or only in tumor core using tissue microarrays) on top of different characteristics of the study cohort. Several authors also reported discordant results to ours when compared CRC patients with synchronous LM vs. stage I–III patients [65]. However, direct comparisons of these to our metachronous cohort would be incorrect because in our study design all patients from the metachronous group indeed developed LM after resection of pCRC, supporting the hypothesis that micrometastases may have already been present in the liver or lymph nodes.

### 4.5 Limitations of the Study

The current study is relatively limited by the modest sample size, but this does not affect the statistically robust results. Earlier studies describing abundance and prognostic significance of T cells between pCRC and LM had comparable [30, 31] or even smaller group sizes [28]. Moreover, the study design of evaluating tumor‐infiltrating T cells in paired samples of pCRC and LM in both synchronous and metachronous disease is novel. Nevertheless, observed opposite effects of the Foxp3+/CD4+ ratio in synchronous vs. metachronous disease require validation in a larger cohort. Because of the relatively small group size, it was difficult to find robust associations between cell densities and response to chemotherapy. Nevertheless, evaluation of tumor‐infiltrating Foxp3+ and CD4+ cells in CRC patients has promise to serve as another important parameter for defining the timing of oncological treatment.

The choice of a single Foxp3 and CD4 as markers for characterizing Tregs and T helper populations can be debated as not depicting their polarization state; however, both are critical to the development of these cells and are currently the most frequently utilized markers for them.

Analyzing multiple sections from primary could better capture intratumoral heterogeneity and may provide additional insights in future studies. Also, immune infiltrates were only examined in a single metastatic lesion per patient; however, the least‐infiltrated LM, assumed to hold the strongest prognostic significance [40], were selected. Results would be further enhanced by integration with other immune cell populations and whole exome sequencing data, which is underway in our laboratory.

## 5 Conclusion

To the best of our knowledge this is the first study directly comparing the distribution and the prognostic significance of CD4+ and Foxp3+ cells between pCRC and LM between synchronous and metachronous disease. Our study design allowed us to demonstrate the prognostic importance of interactions between CD4+ and Foxp3+ cells in the primary tumor. The high Foxp3+/CD4+ cells ratio was associated with shorter survival in metachronous and longer survival in synchronous disease. Foxp3+/CD4+ cells ratio in pCRC may help better stratify CRC patients with synchronous LM after resection of primary tumor. The present results complement the earlier evidence on CD8+ and CD3+ on the role of T cells in modulating CRC outcome.

## Author Contributions

**Esraa Ali:** data curation, formal analysis. **Sergii Pavlov:** data curation, formal analysis. **Václav Liška:** conceptualization, validation, writing – review and editing, resources, project administration, funding acquisition. **Ondřej Daum:** data curation, methodology, validation, writing – review and editing. **Lenka Červenková:** data curation, methodology, formal analysis. **Andriy Trailin:** data curation, methodology, validation, formal analysis, writing – original draft. **Ondřej Vyčítal:** data curation, methodology, formal analysis. **Wenjing Ye:** data curation, formal analysis. **Kari Hemminki:** conceptualization, validation, writing – review and editing, resources, supervision, funding acquisition. **Miroslav Jiřík:** data curation, formal analysis. **Filip Ambrozkiewicz:** data curation, methodology, formal analysis.

## Funding

This research was funded by the grants AZV NU21‐03‐00506 and AZV NW24‐03‐00521.

## Ethics Statement

This retrospective study was conducted in compliance with the ethical standards outlined in the Declaration of Helsinki (2013 version). The need for informed consent was waived by the Ethics Committee of the Faculty of Medicine and University Hospital in Pilsen. The study was approved by the Ethics Committee of the Faculty of Medicine and University Hospital in Pilsen (300/2020, 17 June 2020).

## Consent

The authors have nothing to report.

## Conflicts of Interest

The authors declare no conflicts of interest.

## Supporting information


**Figure S1:** (A) Flow chart of sample selection; (B) QuPath program window showing annotated images after immunohistochemical staining for CD4 and Foxp3; (C) Regions and groups compared (arrows) in the statistical analysis. Abbreviations: CRC: colorectal cancer; LM: liver metastases; pCRC: primary colorectal cancer; TC: tumor center; IM: inner margin; OM: outer margin; PT: peritumor zone.
**Figure S2:** Survival analysis since colon surgery. By the end of follow‐up 42 (76.4%) and 24 (54.5%) patients had died in synchronous and in metachronous group, respectively.
**Figure S3:** Survival analysis since liver surgery.
**Figure S4:** Representative immunostaining of CD4+ and Foxp3+ cells in the invasive margin of primary colorectal cancer (A) and liver metastases of colorectal cancer (B). Foxp3 cells (brown nucleus, DAB chromogen) and CD4 cells (red cytoplasm, AEC chromogen). Organized aggregates of lymphocytes are visible on the border of tumor and non‐tumor tissue. Objective 20×.
**Figure S5:** Kaplan–Meier analysis for DFS according to above median vs. below median inner margin to outer margin ratio of Foxp3+ cells in LM of CRC patients with metachronous LM. *p* value according to log‐rank test.
Abbreviations: DFS: disease‐free survival, CRC: colorectal cancer, LM: liver metastases.
**Table S1:** Spearman correlation between T cells in primary and metastatic sites in CRC patients.
**Table S2:** Spearman correlation between CD4+ and Foxp3+ T cells in primary tumor and in LM in CRC patients.
**Table S3:** Hazard ratios for DFS between High vs. Low T cell density in pCRC and LM in CRC patients with synchronous and metachronous metastases.
**Table S4:** Hazard ratios for TTR between High vs. Low T cell density in pCRC and LM in CRC patients with synchronous and metachronous metastases.
**Table S5:** Hazard ratios for OS between High vs. Low T cell density in pCRC and LM in CRC patients with synchronous and metachronous metastases.
**Table S6:** Hazard ratios for TTR between above median vs. under median Foxp3 to CD4 ratio of T cell density per individual ROI in pCRC and LM in CRC patients with synchronous and metachronous metastases.
**Table S7:** Hazard ratios for OS between above median vs. under median Foxp3 to CD4 ratio of T cell density per individual ROI in pCRC and LM in CRC patients with synchronous and metachronous metastases.
**Table S8:** Hazard ratios for DFS between above median vs. under median Foxp3 to CD4 ratio of T cell density per individual ROI in LM in CRC patients with synchronous and metachronous metastases.
**Table S9:** Hazard ratios for DFS, TTR and OS between above median vs. below median inner margin to outer margin ratio of T cell density in pCRC and LM in CRC patients with synchronous and metachronous metastases.
**Table S10:** Associations of clinical and pathology variables in pCRC and LM with TTR (univariable analysis) in CRC patients with synchronous and metachronous metastases.
**Table S11:** Hazard ratios for DFS and TTR between above median vs. below median Foxp3/CD4 ratio in OM of pCRC and IM/OM ratio of Foxp3+ T cell in LM of metachronous group* (multivariable analysis).
**Table S12:** Association between timing of chemotherapy and outcomes in CRC patients with synchronous and metachronous metastases.
**Table S13:** Association between immune cells and clinical and pathological variables in CRC patients with synchronous and metachronous metastases.
**Table S14:** Association between immune cells and chemotherapy before and after liver resection in CRC patients with synchronous and metachronous metastases.
**Table S15:** Association between immune cells and FOLFOX‐based chemotherapy versus other in CRC patients with synchronous and metachronous metastases.

## Data Availability

The data that support the findings of this study are available from the corresponding author upon reasonable request.
