# Supplementary material for: Foxp3+/CD4+ Cell Ratio in Primary Colorectal Cancer Predicts Opposite Prognoses Following Resection of Synchronous or Metachronous Liver Metastases
Source: Cancer Med. 2026 May 29;15(6):e71989. doi: 10.1002/cam4.71989 (PMC13238659; doi:10.1002/cam4.71989)
Supplement: Supplementary file 1 — Figure S1: (A) Flow chart of sample selection; (B) QuPath program window showing annotated images after immunohistochemical staining for CD4 and Foxp3; (C) Regions and groups compared (arrows) in the statistical analysis. Abbreviations: CRC: colorectal cancer; LM: liver metastases; pCRC: primary colorectal cancer; TC: tumor center; IM: inner margin; OM: outer margin; PT: peritumor zone. Figure S2: Survival analysis since colon surgery. By the end of follow‐up 42 (76.4%) and 24 (54.5%) patients had died in synchronous and in metachronous group, respectively. Figure S3: Survival analysis since liver surgery. Figure S4: Representative immunostaining of CD4+ and Foxp3+ cells in the invasive margin of primary colorectal cancer (A) and liver metastases of colorectal cancer (B). Foxp3 cells (brown nucleus, DAB chromogen) and CD4 cells (red cytoplasm, AEC chromogen). Organized aggregates of lymphocytes are visible on the border of tumor and non‐tumor tissue. Objective 20×. Figure S5: Kaplan–Meier analysis for DFS according to above median vs. below median inner margin to outer margin ratio of Foxp3+ cells in LM of CRC patients with metachronous LM. p value according to log‐rank test. Abbreviations: DFS: disease‐free survival, CRC: colorectal cancer, LM: liver metastases. Table S1: Spearman correlation between T cells in primary and metastatic sites in CRC patients. Table S2: Spearman correlation between CD4+ and Foxp3+ T cells in primary tumor and in LM in CRC patients. Table S3: Hazard ratios for DFS between High vs. Low T cell density in pCRC and LM in CRC patients with synchronous and metachronous metastases. Table S4: Hazard ratios for TTR between High vs. Low T cell density in pCRC and LM in CRC patients with synchronous and metachronous metastases. Table S5: Hazard ratios for OS between High vs. Low T cell density in pCRC and LM in CRC patients with synchronous and metachronous metastases. Table S6: Hazard ratios for TTR between above median vs. under median Foxp3 to [file CAM4-15-e71989-s001.docx]

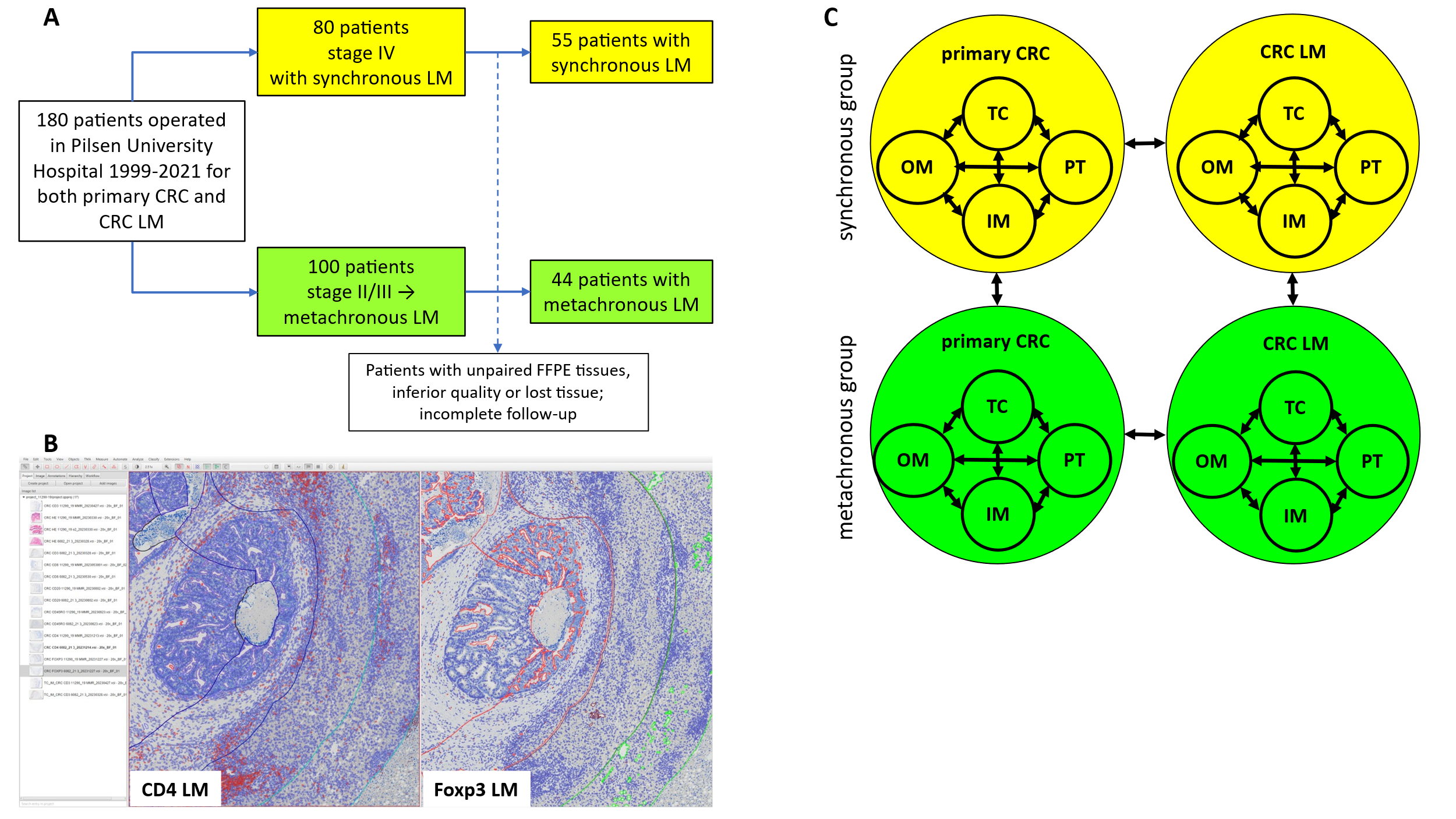


**Figure S1.** (A) Flow chart of sample selection; (B) QuPath program window showing annotated images after immunohistochemical staining for CD4 and Foxp3; (C) Regions and groups compared (arrows) in the statistical analysis. Abbreviations: CRC: colorectal cancer; LM: liver metastases; pCRC: primary colorectal cancer; TC: tumor center; IM: inner margin; OM: outer margin; PT: peritumor zone.


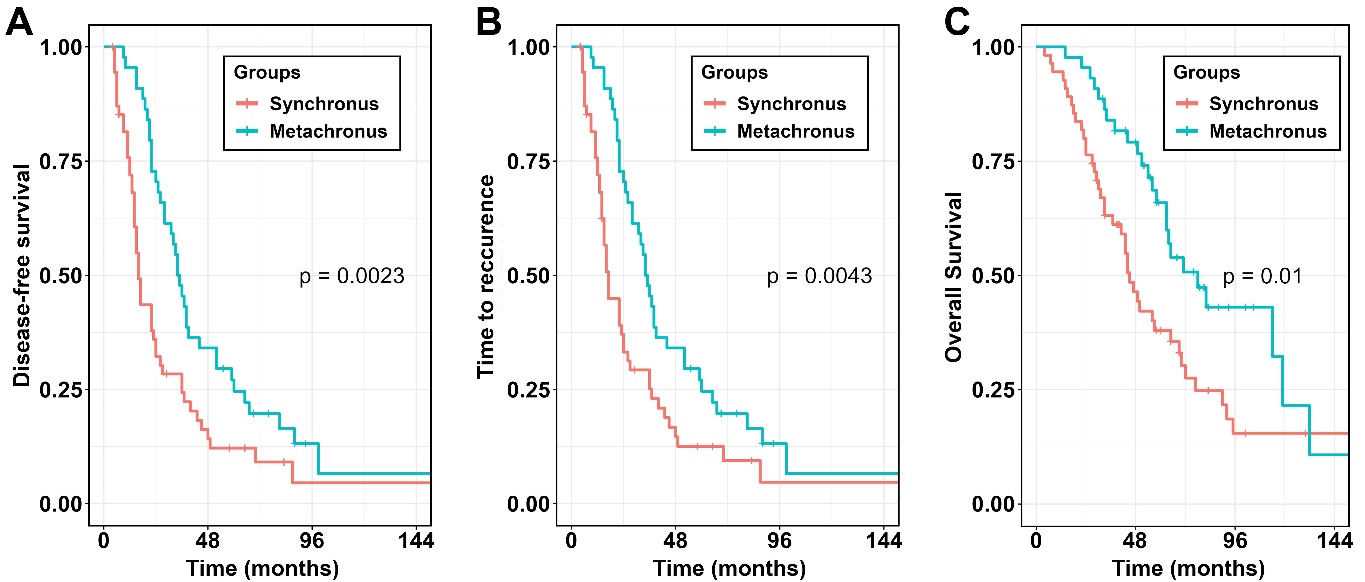


**Figure S2**. Survival analysis since colon surgery. By the end of follow-up 42 (76.4%) and 24 (54.5%) patients had died in synchronous and in metachronous group, respectively.


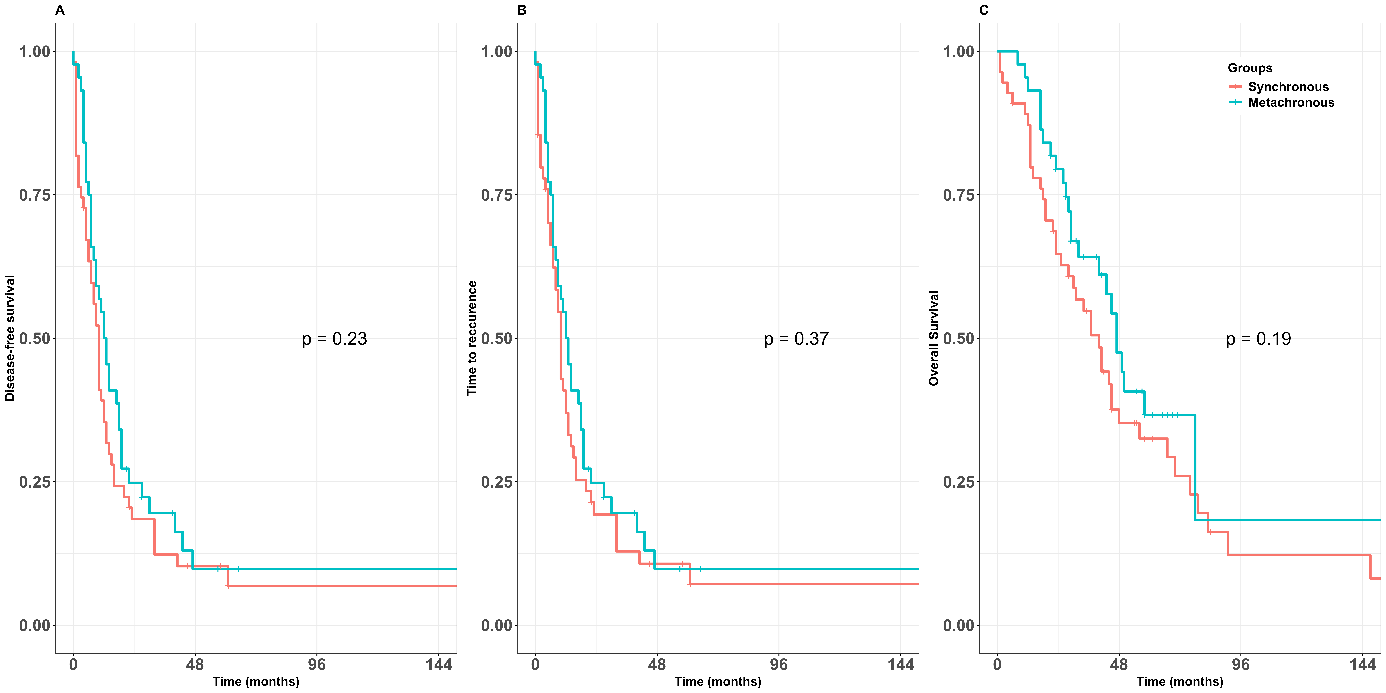


**Figure S3**. Survival analysis since liver surgery.


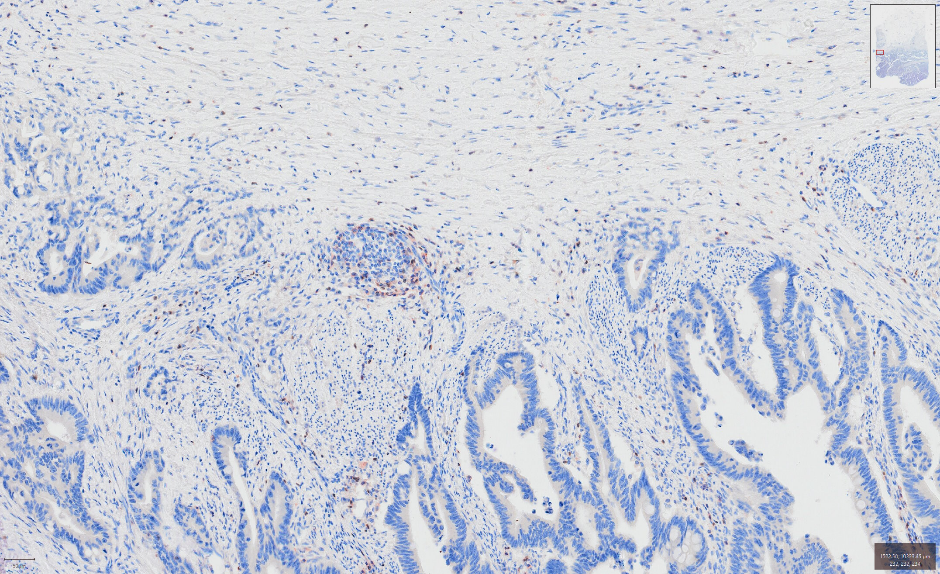

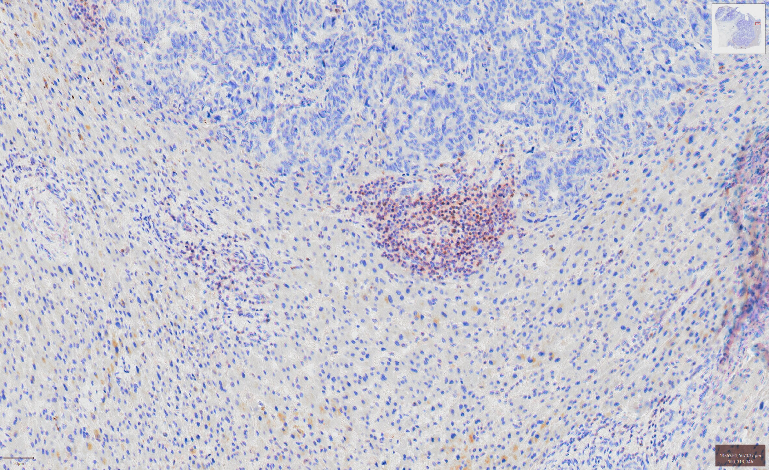


**40 μm**

**40 μm**

**A**

**B**

**Figure S4.** Representative immunostaining of CD4+ and Foxp3+ cells in the invasive margin of primary colorectal cancer (A) and liver metastases of colorectal cancer (B). Foxp3 cells (brown nucleus, DAB chromogen) and CD4 cells (red cytoplasm, AEC chromogen). Organized aggregates of lymphocytes are visible on the border of tumor and non-tumor tissue. Objective 20×.


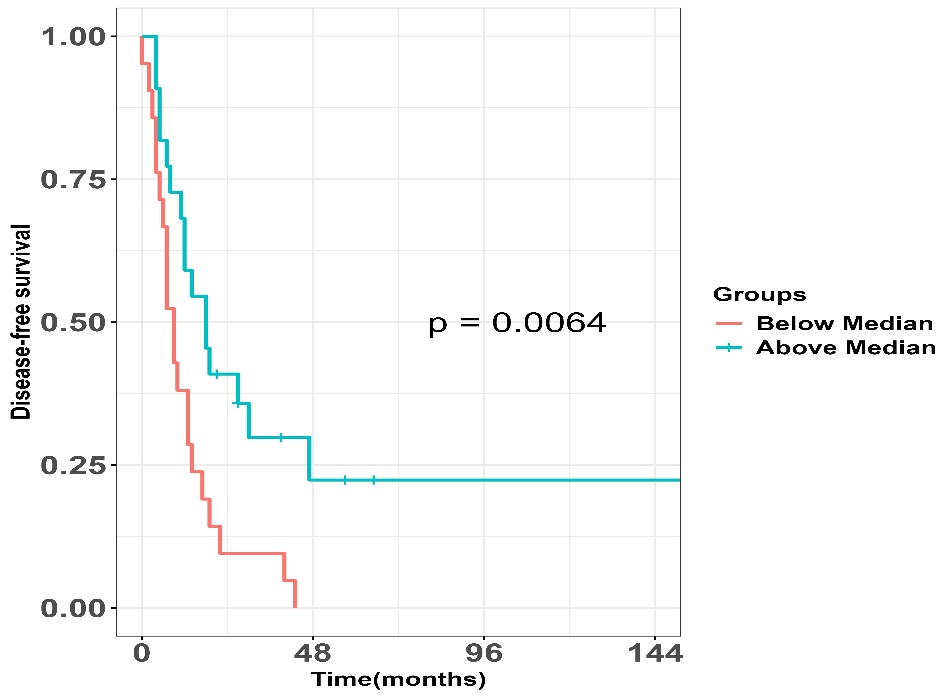


**Figure S5.** Kaplan–Meier analysis for DFS according to above median vs. below median inner margin to outer margin ratio of Foxp3+ cells in LM of CRC patients with metachronous LM. P value according to log-rank test.

Abbreviations: DFS: disease-free survival, CRC: colorectal cancer, LM: liver metastases.

**Supplementary material and methods**

Protocol for sequential IHC staining for CD4 and FOXP3

1. Heat-induced epitope retrieval using BOND Epitope Retrieval Solution 2 for 20 minute (AR9640)
2. Staining protocol: Modified *IHC Protocol F - Autostainer BOND RXm (Leica Biosystems)
   1. Peroxidase Block; 5 min; component of BOND Polymer Refine Detection (DS9800)
   2. MARKER; 15 min; BOND Ready-to-Use Primary Antibody CD4; clone 4B12 (PA0427)
   3. Post Primary Antibody; 8 min; component of BOND Polymer Refine Detection (DS9800)
   4. Polymer; 8 min; component of BOND Polymer Refine Detection (DS9800)
   5. AEC Chromogen Concentrate 925804 Biolegend; 10 min
   6. Hematoxylin; 5 min; component of BOND Polymer Refine Detection (DS9800)
3. Mounting in glycergel (Dako Glycergel, Mounting Medium, Code C0563)
4. Scanning
5. Removal of coverslips, warm distilled water (50°C), overnight
6. AEC washout
   1. 70% alcohol; 30 sec
   2. 80% alcohol; 30 sec
   3. 96% alcohol; 5 min
   4. 80% alcohol; 30 sec
   5. 70% alcohol; 30 sec
   6. distilled water; 5 min
7. Heat-induced epitope retrieval using BOND Epitope Retrieval Solution 2 for 40 minute (AR9640) = STRIPING of previous antibodies
8. Staining protocol: *IHC Protocol F - Autostainer BOND RXm (Leica Biosystems)
   1. Peroxide Block; 5 min; component of BOND Polymer Refine Detection (DS9800)
   2. MARKER; 15 min; BOND Ready-to-Use Primary Antibody FOXP3, clone 236A/E7) (PA0263)
   3. Post Primary Antibody; 8 min; component of BOND Polymer Refine Detection (DS9800)
   4. Polymer; 8 min; component of BOND Polymer Refine Detection (DS9800)
   5. Mixed DAB Refine; 10 min; component of BOND Polymer Refine Detection (DS9800)
   6. Hematoxylin; 5 min; component of BOND Polymer Refine Detection (DS9800)
9. Dehydratation, clearing and mounting

Comment: rinsing steps are omitted

**Supplementary tables**

**Table S1.** Spearman correlation between T cells in primary and metastatic sites in CRC patients.

| pCRC | LM | | | | | |
| --- | --- | --- | --- | --- | --- | --- |
|  | synchronous | | | | | |
|  | **CD4** | TC | IM | OM | | PT |
|  | TC | 0.32^&,^* | 0.36* | 0.42** | | 0.46** |
|  | IM | 0.31* | 0.39** | 0.40** | | 0.42** |
|  | OM | 0.27 | 0.41** | 0.32* | | 0.42** |
|  | PT | 0.24 | 0.33* | 0.34* | | 0.49*** |
|  | **Foxp3** |  |  |  | |  |
|  | TC | 0.42** | 0.30* | 0.33* | | 0.16 |
|  | IM | 0.43** | 0.42** | 0.34* | | 0.06 |
|  | OM | 0.27 | 0.35* | 0.26 | | 0.13 |
|  | PT | 0.14 | 0.25 | 0.14 | | -0.09 |
|  | metachronous | | | | | |
|  | **CD4** | TC | IM | OM | PT | |
|  | TC | 0.11 | 0.15 | 0.24 | 0.16 | |
|  | IM | 0.29 | 0.30 | 0.30 | 0.13 | |
|  | OM | 0.22 | 0.26 | 0.39* | 0.27 | |
|  | PT | 0.27 | 0.28 | 0.36* | 0.23 | |
|  | **Foxp3** |  |  |  |  | |
|  | TC | 0.41** | 0.40* | 0.37* | 0.33* | |
|  | IM | 0.49** | 0.51*** | 0.49** | 0.45** | |
|  | OM | 0.36* | 0.40* | 0.40* | 0.36* | |
|  | PT | 0.30 | 0.40* | 0.39* | 0.28 | |
|  |  |  |  |  |  |  |

Notes: ^&^: Spearman's ρ, *: for Spearman's ρ <0.05, **: for Spearman's ρ <0.01, ***: for Spearman's ρ <0.001.

Abbreviations: CRC: colorectal cancer; LM: liver metastases; pCRC: primary colorectal cancer; TC: tumor center; IM: inner margin; OM: outer margin; PT: peritumor zone.

**Table S2.** Spearman correlation between CD4+ and Foxp3+ T cells in primary tumor and in LM in CRC patients

| CD4 pCRC | Foxp3 pCRC | | | | | | | | | | |
| --- | --- | --- | --- | --- | --- | --- | --- | --- | --- | --- | --- |
|  | synchronous | | | | | | | | | | |
|  |  | TC | | IM | | OM | | | | | PT |
|  | TC | 0.58^&,^*** | | 0.44** | | 0.41** | | | | | 0.27 |
|  | IM | 0.47*** | | 0.50*** | | 0.52*** | | | | | 0.35* |
|  | OM | 0.07 | | 0.16 | | 0.44** | | | | | 0.29* |
|  | PT | 0.14 | | 0.10 | | 0.39** | | | | | 0.37** |
|  | metachronous | | | | | | | | | | |
|  |  | TC | | IM | | OM | | PT | | | |
|  | TC | 0.51*** | | 0.56*** | | 0.45** | | 0.46** | | | |
|  | IM | 0.57*** | | 0.64*** | | 0.50** | | 0.45** | | | |
|  | OM | 0.46** | | 0.51*** | | 0.50** | | 0.52*** | | | |
|  | PT | 0.47** | | 0.51*** | | 0.55*** | | 0.62*** | | | |
| CD4 LM | Foxp3 LM | | | | | | | | | | |
|  | synchronous | | | | | | | | | | |
|  |  | | TC | | IM | | OM | | | PT | |
|  | TC | | 0.76*** | | 0.69*** | | 0.48*** | | | 0.29* | |
|  | IM | | 0.69*** | | 0.71*** | | 0.42** | | | 0.27 | |
|  | OM | | 0.46*** | | 0.45** | | 0.51*** | | | 0.34* | |
|  | PT | | 0.41** | | 0.42** | | 0.51*** | | | 0.48*** | |
|  | metachronous | | | | | | | | | | |
|  |  | | TC | | IM | | OM | | PT | | |
|  | TC | | 0.68*** | | 0.66*** | | 0.60*** | | 0.45** | | |
|  | IM | | 0.68*** | | 0.77*** | | 0.69*** | | 0.45** | | |
|  | OM | | 0.47** | | 0.53*** | | 0.70*** | | 0.40** | | |
|  | PT | | 0.46** | | 0.42** | | 0.59*** | | 0.43** | | |

Notes: ^&^: Spearman's ρ, *: for Spearman's ρ <0.05, **: for Spearman's ρ <0.01, ***: for Spearman's ρ <0.001.

Abbreviations: CRC: colorectal cancer; LM: liver metastases; pCRC: primary colorectal cancer; TC: tumor center; IM: inner margin; OM: outer margin; PT: peritumor zone.

**Table S3.** Hazard ratios for DFS between High vs Low T cell density in pCRC and LM in CRC patients with synchronous and metachronous metastases

| primary CRC | | | | | | |
| --- | --- | --- | --- | --- | --- | --- |
| Cell type/  location | Synchronous | | | Metachronous | | |
|  | Group | N (%) | HR | Group | N (%) | HR |
| CD4 TC | Low | 16 (30.8) | - | Low | 10 (24.4) | - |
|  | High | 36 (69.2) | 0.78 (0.42-1.43, p=0.420) | High | 31 (75.6) | 0.67 (0.32-1.41, p=0.287) |
| CD4 IM | Low | 13 (25.5) | - | Low | 11 (26.8) | - |
|  | High | 38 (74.5) | 0.92 (0.48-1.76, p=0.809) | High | 30 (73.2) | 0.66 (0.32-1.38, p=0.269) |
| CD4 OM | Low | 12 (23.5) | - | Low | 10 (24.4) | - |
|  | High | 39 (76.5) | 1.30 (0.65-2.58, p=0.455) | High | 31 (75.6) | 0.65 (0.31-1.38, p=0.262) |
| CD4 PT | Low | 12 (23.5) | - | Low | 10 (24.4) | - |
|  | High | 39 (76.5) | 1.34 (0.67-2.66, p=0.410) | High | 31 (75.6) | 0.73 (0.34-1.54, p=0.404) |
| foxp3 TC | Low | 13 (24.5) | - | Low | 11 (27.5) | - |
|  | High | 40 (75.5) | 0.69 (0.36-1.31, p=0.256) | High | 29 (72.5) | 1.79 (0.82-3.91, p=0.145) |
| foxp3 IM | Low | 13 (25.5) | - | Low | 11 (27.5) | - |
|  | High | 38 (74.5) | 0.66 (0.34-1.25, p=0.201) | High | 29 (72.5) | 1.56 (0.73-3.36, p=0.252) |
| foxp3 OM | Low | 13 (25.5) | - | Low | 10 (25.0) | - |
|  | High | 38 (74.5) | 0.61 (0.31-1.18, p=0.144) | High | 30 (75.0) | 0.89 (0.43-1.87, p=0.764) |
| foxp3 PT | Low | 13 (25.5) | - | Low | 11 (27.5) | - |
|  | High | 38 (74.5) | 0.74 (0.38-1.43, p=0.368) | High | 29 (72.5) | 0.88 (0.43-1.81, p=0.727) |
| LM | | | | | | |
| CD4 TC | Low | 13 (26.0) | - | Low | 11 (26.2) | - |
|  | High | 37 (74.0) | 1.37 (0.67-2.78, p=0.386) | High | 31 (73.8) | 0.64 (0.31-1.32, p=0.225) |
| CD4 IM | Low | 13 (26.0) | - | Low | 11 (26.2) | - |
|  | High | 37 (74.0) | 1.01 (0.52-1.96, p=0.983) | High | 31 (73.8) | 1.20 (0.58-2.49, p=0.620) |
| CD4 OM | Low | 13 (25.5) | - | Low | 11 (26.2) | - |
|  | High | 38 (74.5) | 1.60 (0.80-3.19, p=0.180) | High | 31 (73.8) | 0.78 (0.37-1.63, p=0.506) |
| CD4 PT | Low | 14 (27.5) | - | Low | 10 (24.4) | - |
|  | High | 37 (72.5) | 1.13 (0.59-2.19, p=0.710) | High | 31 (75.6) | 0.55 (0.26-1.16, p=0.117) |
| foxp3 TC | Low | 13 (25.5) | - | Low | 11 (25.6) | - |
|  | High | 38 (74.5) | 0.77 (0.39-1.53, p=0.453) | High | 32 (74.4) | 0.91 (0.43-1.90, p=0.794) |
| foxp3 IM | Low | 13 (25.5) | - | Low | 11 (25.6) | - |
|  | High | 38 (74.5) | 0.77 (0.40-1.50, p=0.450) | High | 32 (74.4) | 0.73 (0.35-1.52, p=0.404) |
| foxp3 OM | Low | 14 (27.5) | - | Low | 11 (25.6) | - |
|  | High | 37 (72.5) | 0.87 (0.46-1.65, p=0.671) | High | 32 (74.4) | 0.96 (0.46-2.01, p=0.920) |
| foxp3 PT | Low | 12 (23.5) | - | Low | 11 (26.2) | - |
|  | High | 39 (76.5) | 0.77 (0.39-1.53, p=0.461) | High | 31 (73.8) | 1.27 (0.59-2.72, p=0.540) |

Raw densities of Foxp3+ and CD4+ T cells per area of ROI (mm^2^) were converted into high (25-100) vs low percentile groups. Hazard ratios show the relative risk compared with 1.00 for low group.

Abbreviations: DFS, disease-free survival; ROI, region of interest; CRC: colorectal cancer; pCRC: primary colorectal cancer; LM: liver metastases; HR: hazard ratio; CI: confidence interval; TC: tumor center; IM: inner margin; OM: outer margin; PT: peritumor zone.

**Table S4**. Hazard ratios for TTR between High vs Low T cell density in pCRC and LM in CRC patients with synchronous and metachronous metastases

| primary CRC | | | | | | |
| --- | --- | --- | --- | --- | --- | --- |
| Cell type/  location | Synchronous | | | Metachronous | | |
|  | Group | N (%) | HR | Group | N (%) | HR |
| CD4 TC | Low | 16 (30.8) | - | Low | 10 (24.4) | - |
|  | High | 36 (69.2) | 0.80 (0.43-1.50, p=0.490) | High | 31 (75.6) | 0.67 (0.32-1.41, p=0.287) |
| CD4 IM | Low | 13 (25.5) | - | Low | 11 (26.8) | - |
|  | High | 38 (74.5) | 0.97 (0.50-1.90, p=0.941) | High | 30 (73.2) | 0.66 (0.32-1.38, p=0.269) |
| CD4 OM | Low | 12 (23.5) | - | Low | 10 (24.4) | - |
|  | High | 39 (76.5) | 1.23 (0.62-2.46, p=0.552) | High | 31 (75.6) | 0.65 (0.31-1.38, p=0.262) |
| CD4 PT | Low | 12 (23.5) | - | Low | 10 (24.4) | - |
|  | High | 39 (76.5) | 1.27 (0.63-2.53, p=0.506) | High | 31 (75.6) | 0.73 (0.34-1.54, p=0.404) |
| foxp3 TC | Low | 13 (24.5) | - | Low | 11 (27.5) | - |
|  | High | 40 (75.5) | 0.64 (0.33-1.22, p=0.174) | High | 29 (72.5) | 1.79 (0.82-3.91, p=0.145) |
| foxp3 IM | Low | 13 (25.5) | - | Low | 11 (27.5) | - |
|  | High | 38 (74.5) | 0.61 (0.32-1.17, p=0.135) | High | 29 (72.5) | 1.56 (0.73-3.36, p=0.252) |
| foxp3 OM | Low | 13 (25.5) | - | Low | 10 (25.0) | - |
|  | High | 38 (74.5) | 0.64 (0.32-1.27, p=0.198) | High | 30 (75.0) | 0.89 (0.43-1.87, p=0.764) |
| foxp3 PT | Low | 13 (25.5) | - | Low | 11 (27.5) | - |
|  | High | 38 (74.5) | 0.68 (0.35-1.33, p=0.259) | High | 29 (72.5) | 0.88 (0.43-1.81, p=0.727) |
| LM | | | | | | |
| CD4 TC | Low | 13 (26.0) | - | Low | 11 (26.2) | - |
|  | High | 37 (74.0) | 1.49 (0.71-3.13, p=0.289) | High | 31 (73.8) | 0.64 (0.31-1.32, p=0.225) |
| CD4 IM | Low | 13 (26.0) | - | Low | 11 (26.2) | - |
|  | High | 37 (74.0) | 1.08 (0.54-2.15, p=0.834) | High | 31 (73.8) | 1.20 (0.58-2.49, p=0.620) |
| CD4 OM | Low | 13 (25.5) | - | Low | 11 (26.2) | - |
|  | High | 38 (74.5) | 1.53 (0.76-3.06, p=0.231) | High | 31 (73.8) | 0.78 (0.37-1.63, p=0.506) |
| CD4 PT | Low | 14 (27.5) | - | Low | 10 (24.4) | - |
|  | High | 37 (72.5) | 1.07 (0.55-2.08, p=0.847) | High | 31 (75.6) | 0.55 (0.26-1.16, p=0.117) |
| foxp3 TC | Low | 13 (25.5) | - | Low | 11 (25.6) | - |
|  | High | 38 (74.5) | 0.81 (0.40-1.66, p=0.569) | High | 32 (74.4) | 0.91 (0.43-1.90, p=0.794) |
| foxp3 IM | Low | 13 (25.5) | - | Low | 11 (25.6) | - |
|  | High | 38 (74.5) | 0.82 (0.41-1.62, p=0.562) | High | 32 (74.4) | 0.73 (0.35-1.52, p=0.404) |
| foxp3 OM | Low | 14 (27.5) | - | Low | 11 (25.6) | - |
|  | High | 37 (72.5) | 0.92 (0.47-1.78, p=0.795) | High | 32 (74.4) | 0.96 (0.46-2.01, p=0.920) |
| foxp3 PT | Low | 12 (23.5) | - | Low | 11 (26.2) | - |
|  | High | 39 (76.5) | 0.96 (0.46-2.00, p=0.910) | High | 31 (73.8) | 1.27 (0.59-2.72, p=0.540) |

Raw densities of Foxp3+ and CD4+ T cells per area of ROI (mm2) were converted into high (25-100) vs low percentile groups. Hazard ratios show the relative risk compared with 1.00 for low group.

Abbreviations: TTR, time to recurrence; ROI, region of interest; CRC: colorectal cancer; pCRC: primary colorectal cancer; LM: liver metastases; HR: hazard ratio; CI: confidence interval; TC: tumor center; IM: inner margin; OM: outer margin; PT: peritumor zone.

**Table S5.** Hazard ratios for OS between High vs Low T cell density in pCRC and LM in CRC patients with synchronous and metachronous metastases

| primary CRC | | | | | | |
| --- | --- | --- | --- | --- | --- | --- |
| Cell type/  location | Synchronous | | | Metachronous | | |
|  | Group | N (%) | HR | Group | N (%) | HR |
| CD4 TC | Low | 16 (30.8) | - | Low | 10 (24.4) | - |
|  | High | 36 (69.2) | 0.56 (0.29-1.08, p=0.085) | High | 31 (75.6) | 0.48 (0.21-1.13, p=0.093) |
| CD4 IM | Low | 13 (25.5) | - | Low | 11 (26.8) | - |
|  | High | 38 (74.5) | 0.73 (0.36-1.45, p=0.363) | High | 30 (73.2) | 0.60 (0.26-1.41, p=0.244) |
| CD4 OM | Low | 12 (23.5) | - | Low | 10 (24.4) | - |
|  | High | 39 (76.5) | 0.88 (0.42-1.85, p=0.742) | High | 31 (75.6) | 0.69 (0.28-1.71, p=0.426) |
| CD4 PT | Low | 12 (23.5) | - | Low | 10 (24.4) | - |
|  | High | 39 (76.5) | 0.84 (0.40-1.77, p=0.643) | High | 31 (75.6) | 0.43 (0.18-1.05, p=0.063) |
| foxp3 TC | Low | 13 (24.5) | - | Low | 11 (27.5) | - |
|  | High | 40 (75.5) | 0.71 (0.33-1.52, p=0.373) | High | 29 (72.5) | 0.96 (0.39-2.36, p=0.926) |
| foxp3 IM | Low | 13 (25.5) | - | Low | 11 (27.5) | - |
|  | High | 38 (74.5) | 0.48 (0.22-1.03, p=0.060) | High | 29 (72.5) | 0.81 (0.33-1.97, p=0.642) |
| foxp3 OM | Low | 13 (25.5) | - | Low | 10 (25.0) | - |
|  | High | 38 (74.5) | 0.66 (0.31-1.39, p=0.276) | High | 30 (75.0) | 0.49 (0.20-1.21, p=0.122) |
| foxp3 PT | Low | 13 (25.5) | - | Low | 11 (27.5) | - |
|  | High | 38 (74.5) | 0.89 (0.43-1.85, p=0.760) | High | 29 (72.5) | 0.50 (0.21-1.21, p=0.124) |
| LM | | | | | | |
| CD4 TC | Low | 13 (26.0) | - | Low | 11 (26.2) | - |
|  | High | 37 (74.0) | 1.59 (0.72-3.54, p=0.254) | High | 31 (73.8) | **0.27 (0.12-0.63, p=0.002)** |
| CD4 IM | Low | 13 (26.0) | - | Low | 11 (26.2) | - |
|  | High | 37 (74.0) | 1.13 (0.54-2.37, p=0.755) | High | 31 (73.8) | 0.63 (0.27-1.50, p=0.299) |
| CD4 OM | Low | 13 (25.5) | - | Low | 11 (26.2) | - |
|  | High | 38 (74.5) | 0.87 (0.44-1.73, p=0.694) | High | 31 (73.8) | 0.52 (0.22-1.24, p=0.139) |
| CD4 PT | Low | 14 (27.5) | - | Low | 10 (24.4) | - |
|  | High | 37 (72.5) | 0.77 (0.39-1.51, p=0.446) | High | 31 (75.6) | 0.64 (0.27-1.53, p=0.313) |
| foxp3 TC | Low | 13 (25.5) | - | Low | 11 (25.6) | - |
|  | High | 38 (74.5) | 1.03 (0.47-2.23, p=0.943) | High | 32 (74.4) | 0.66 (0.28-1.55, p=0.337) |
| foxp3 IM | Low | 13 (25.5) | - | Low | 11 (25.6) | - |
|  | High | 38 (74.5) | 1.16 (0.55-2.42, p=0.701) | High | 32 (74.4) | 0.62 (0.26-1.46, p=0.269) |
| foxp3 OM | Low | 14 (27.5) | - | Low | 11 (25.6) | - |
|  | High | 37 (72.5) | 0.77 (0.39-1.51, p=0.450) | High | 32 (74.4) | 0.68 (0.29-1.61, p=0.376) |
| foxp3 PT | Low | 12 (23.5) | - | Low | 11 (26.2) | - |
|  | High | 39 (76.5) | 0.85 (0.41-1.76, p=0.656) | High | 31 (73.8) | 0.80 (0.32-1.96, p=0.621) |

Raw densities of Foxp3+ and CD4+ T cells per area of ROI (mm2) were converted into high (25-100) vs low percentile groups. Hazard ratios show the relative risk compared with 1.00 for low group.

Abbreviations: OS: overall survival; ROI, region of interest; CRC: colorectal cancer; pCRC: primary colorectal cancer; LM: liver metastases; HR: hazard ratio; CI: confidence interval; TC: tumor center; IM: inner margin; OM: outer margin; PT: peritumor zone.

**Table S6**. Hazard ratios for TTR between above median vs under median Foxp3 to CD4 ratio of T cell density per individual ROI in pCRC and LM in CRC patients with synchronous and metachronous metastases

| FoxP3/CD4 ratio pCRC | | | | | | |
| --- | --- | --- | --- | --- | --- | --- |
| Cell type/location | Synchronous | | | Metachronous | | |
|  | Group | N (%) | HR | Group | N (%) | HR |
| foxp3/cd4 TC | Below | 26 (50.0) | - | Below | 20 (50.0) | - |
|  | Above | 26 (50.0) | 1.36 (0.74-2.48, p=0.322) | Above | 20 (50.0) | 1.85 (0.92-3.71, p=0.082) |
| foxp3/cd4 IM | Below | 25 (49.0) | - | Below | 20 (50.0) | - |
|  | Above | 26 (51.0) | 0.61 (0.33-1.13, p=0.117) | Above | 20 (50.0) | 1.09 (0.56-2.15, p=0.795) |
| foxp3/cd4 OM | Below | 25 (49.0) | - | Below | 20 (50.0) | - |
|  | Above | 26 (51.0) | **0.52 (0.27-0.97, p=0.040)** | Above | 20 (50.0) | **2.34 (1.14-4.79, p=0.020)** |
| foxp3/cd4 PT | Below | 25 (49.0) | - | Below | 18 (45.0) | - |
|  | Above | 26 (51.0) | **0.44 (0.23-0.84, p=0.013)** | Above | 22 (55.0) | 1.82 (0.89-3.70, p=0.101) |
| FoxP3/CD4 ratio LM | | | | | | |
| foxp3/cd4 TC | Below | 25 (50.0) | - | Below | 21 (50.0) | - |
|  | Above | 25 (50.0) | 0.95 (0.52-1.75, p=0.877) | Above | 21 (50.0) | 1.19 (0.61-2.31, p=0.615) |
| foxp3/cd4 IM | Below | 25 (50.0) | - | Below | 21 (50.0) | - |
|  | Above | 25 (50.0) | 0.87 (0.48-1.60, p=0.660) | Above | 21 (50.0) | 1.23 (0.63-2.40, p=0.548) |
| foxp3/cd4 OM | Below | 25 (49.0) | - | Below | 22 (52.4) | - |
|  | Above | 26 (51.0) | 1.13 (0.62-2.06, p=0.691) | Above | 20 (47.6) | 1.33 (0.69-2.57, p=0.387) |
| foxp3/cd4 PT | Below | 23 (46.0) | - | Below | 20 (48.8) | - |
|  | Above | 27 (54.0) | 0.81 (0.44-1.48, p=0.485) | Above | 21 (51.2) | 1.74 (0.89-3.40, p=0.103) |

Raw densities of Foxp3+ and CD4+ T cells per area of ROI (mm^2^) were used to calculate FoxP3/CD4 ratio and then categorize patients into above and below median groups. Hazard ratios show the relative risk compared with 1.00 for below median group.

Notes: Bold values indicate statistical significance at the p < 0.05 level.

Abbreviations: TTR: time to recurrence; ROI, region of interest; CRC: colorectal cancer; pCRC: primary colorectal cancer; HR: hazard ratio; CI: confidence interval; TC: tumor center; IM: inner margin; OM: outer margin; PT: peritumor zone.

**Table S7**. Hazard ratios for OS between above median vs under median Foxp3 to CD4 ratio of T cell density per individual ROI in pCRC and LM in CRC patients with synchronous and metachronous metastases

| FoxP3/CD4 ratio pCRC | | | | | | |
| --- | --- | --- | --- | --- | --- | --- |
| Cell type/location | Synchronous | | | Metachronous | | |
|  | Group | N (%) | HR | Group | N (%) | HR |
| foxp3/  cd4 TC | Below | 26 (50.0) | - | Below | 20 (50.0) | - |
|  | Above | 26 (50.0) | 1.35 (0.70-2.58, p=0.368) | Above | 20 (50.0) | 0.98 (0.42-2.28, p=0.963) |
| foxp3/  cd4 IM | Below | 25 (49.0) | - | Below | 20 (50.0) | - |
|  | Above | 26 (51.0) | 0.66 (0.34-1.28, p=0.214) | Above | 20 (50.0) | 0.87 (0.36-2.07, p=0.750) |
| foxp3/  cd4 OM | Below | 25 (49.0) | - | Below | 20 (50.0) | - |
|  | Above | 26 (51.0) | 0.80 (0.42-1.54, p=0.511) | Above | 20 (50.0) | 1.15 (0.49-2.67, p=0.746) |
| foxp3/  cd4 PT | Below | 25 (49.0) | - | Below | 18 (45.0) | - |
|  | Above | 26 (51.0) | 0.81 (0.42-1.55, p=0.518) | Above | 22 (55.0) | 1.07 (0.46-2.51, p=0.875) |
| FoxP3/CD4 ratio LM | | | | | | |
| foxp3/  cd4 TC | Below | 25 (50.0) | - | Below | 21 (50.0) | - |
|  | Above | 25 (50.0) | 0.79 (0.41-1.51, p=0.478) | Above | 21 (50.0) | 1.24 (0.55-2.82, p=0.603) |
| foxp3/  cd4 IM | Below | 25 (50.0) | - | Below | 21 (50.0) | - |
|  | Above | 25 (50.0) | 0.79 (0.41-1.52, p=0.481) | Above | 21 (50.0) | 1.17 (0.52-2.66, p=0.706) |
| foxp3/  cd4 OM | Below | 25 (49.0) | - | Below | 22 (52.4) | - |
|  | Above | 26 (51.0) | 1.38 (0.72-2.66, p=0.332) | Above | 20 (47.6) | 1.12 (0.49-2.56, p=0.787) |
| foxp3/  cd4 PT | Below | 23 (46.0) | - | Below | 20 (48.8) | - |
|  | Above | 27 (54.0) | 1.17 (0.61-2.26, p=0.635) | Above | 21 (51.2) | 1.28 (0.56-2.93, p=0.555) |

Raw densities of Foxp3+ and CD4+ T cells per area of ROI (mm^2^) were used to calculate FoxP3/CD4 ratio and then categorize patients into above and below median groups. Hazard ratios show the relative risk compared with 1.00 for below median group.

Notes: Bold values indicate statistical significance at the p < 0.05 level.

Abbreviations: OS: overall survival; ROI, region of interest; CRC: colorectal cancer; pCRC: primary colorectal cancer; HR: hazard ratio; CI: confidence interval; TC: tumor center; IM: inner margin; OM: outer margin; PT: peritumor zone.

**Table S8**. Hazard ratios for DFS between above median vs under median Foxp3 to CD4 ratio of T cell density per individual ROI in LM in CRC patients with synchronous and metachronous metastases

| FoxP3/CD4 ratio LM | | | | | | |
| --- | --- | --- | --- | --- | --- | --- |
| Cell type/  location | Synchronous | | | Metachronous | | |
|  | Group | N (%) | HR | Group | N (%) | HR |
| foxp3/  cd4 TC | Below | 25 (50.0) | - | Below | 21 (50.0) | - |
|  | Above | 25 (50.0) | 0.87 (0.48-1.57, p=0.633) | Above | 21 (50.0) | 1.19 (0.61-2.31, p=0.615) |
| foxp3/  cd4 IM | Below | 25 (50.0) | - | Below | 21 (50.0) | - |
|  | Above | 25 (50.0) | 0.88 (0.49-1.59, p=0.670) | Above | 21 (50.0) | 1.23 (0.63-2.40, p=0.548) |
| foxp3/  cd4 OM | Below | 25 (49.0) | - | Below | 22 (52.4) | - |
|  | Above | 26 (51.0) | 1.02 (0.57-1.82, p=0.951) | Above | 20 (47.6) | 1.33 (0.69-2.57, p=0.387) |
| foxp3/  cd4 PT | Below | 23 (46.0) | - | Below | 20 (48.8) | - |
|  | Above | 27 (54.0) | 0.73 (0.41-1.32, p=0.305) | Above | 21 (51.2) | 1.74 (0.89-3.40, p=0.103) |

Raw densities of Foxp3+ and CD4+ T cells per area of ROI (mm^2^) were used to calculate FoxP3/CD4 ratio and then categorize patients into above and under median groups. Hazard ratios shows the relative risk compared with 1.00 for under median group.

Notes: Bold values indicate statistical significance at the p < 0.05 level.

Abbreviations: DFS, disease-free survival; ROI, region of interest; LM: liver metastases; CRC: colorectal cancer; LM: liver metastases; HR: hazard ratio; CI: confidence interval; IM: inner margin; OM: outer margin.

**Table S9.** Hazard ratios for DFS, TTR and OS between above median vs below median inner margin to outer margin ratio of T cell density in pCRC and LM in CRC patients with synchronous and metachronous metastases

| Cell type | Synchronous | | | Metachronous | | |
| --- | --- | --- | --- | --- | --- | --- |
|  | Group | N (%) | HR (95% CI), p-value | Group | N (%) | HR (95% CI), p-value |
| **DFS** | | | | | | |
| IM/OM ratio pCRC | | | | | | |
| CD4 | Above median | 26 (51.0) | 1.02 (0.56-1.84), p=0.95 | Above median | 21 (51.2) | 1.37 (0.70-2.70), p=0.36 |
| Foxp3 | Above median | 27 (52.9) | 0.95 (0.53-1.71), p=0.86 | Above median | 20 (50.0) | 1.34 (0.68-2.66), p=0.40 |
| IM/OM ratio LM | | | | | | |
| CD4 | Above median | 25 (50.0) | 0.58 (0.32-1.07), p=0.08 | Above median | 21 (50.0) | 0.88 (0.46-1.69), p=0.70 |
| Foxp3 | Above median | 24 (51.1) | 0.99 (0.54-1.81), p=0.96 | Above median | 22 (51.2) | **0.41 (0.21-0.79), p=0.009** |

| **TTR** | | | | | | |
| --- | --- | --- | --- | --- | --- | --- |
| IM/OM ratio pCRC | | | | | | |
| CD4 | Above median | 26 (51.0) | 1.02 (0.56-1.87), p=0.95 | Above median | 21 (51.2) | 1.37 (0.70-2.70), p=0.36 |
| Foxp3 | Above median | 27 (52.9) | 0.86 (0.47-1.57), p=0.62 | Above median | 20 (50.0) | 1.34 (0.68-2.66), p=0.40 |
| IM/OM ratio LM | | | | | | |
| CD4 | Above median | 25 (50.0) | 0.63 (0.34-1.17), p=0.15 | Above median | 21 (50.0) | 0.88 (0.46-1.69), p=0.70 |
| Foxp3 | Above median | 24 (51.1) | 0.88 (0.47-1.65), p=0.70 | Above median | 22 (51.2) | **0.41 (0.21-0.79), p=0.009** |

| **OS** | | | | | | |
| --- | --- | --- | --- | --- | --- | --- |
| IM/OM ratio pCRC | | | | | | |
| CD4 | Above median | 26 (51.0) | 1.12 (0.57-2.19, p=0.743) | Above median | 21 (51.2) | 0.91 (0.39-2.10, p=0.821) |
| foxp3 | Above median | 27 (52.9) | 1.22 (0.63-2.33, p=0.557) | Above median | 20 (50.0) | 1.42 (0.60-3.36, p=0.425) |
| IM/OM ratio LM | | | | | | |
| CD4 | Above median | 25 (50.0) | 1.05 (0.55-1.99, p=0.890) | Above median | 21 (50.0) | 0.76 (0.34-1.73, p=0.518) |
| foxp3 | Above median | 24 (51.1) | 0.71 (0.36-1.42, p=0.333) | Above median | 22 (51.2) | 0.45 (0.19-1.04, p=0.061) |

Raw densities of CD4+ and Foxp3+ T cells per area of IM and OM (mm^2^) were used to calculate IM to OM ratio and then categorize patients into above and below median groups. Hazard ratios shows the relative risk compared with 1.00 for below median group.

Notes: Bold values indicate statistical significance at the p < 0.05 level.

Abbreviations: DFS, disease-free survival, TTR, time to recurrence; OS: overall survival; ROI, region of interest; LM: liver metastases; CRC: colorectal cancer; pCRC: primary colorectal cancer; HR: hazard ratio; CI: confidence interval; IM: inner margin; OM: outer margin.

**Table S10.** Associations of clinical and pathology variables in pCRC and LM with TTR (univariable analysis) in CRC patients with synchronous and metachronous metastases

| Variables | Group | Synchronous | | Metachronous | |
| --- | --- | --- | --- | --- | --- |
|  |  | N (%) | HR (95% CI), p-value | N (%) | HR (95% CI), p-value |
| Gender | male | 34 (61.8) | 1.00 | 29 (65.9) | 1.00 |
|  | female | 21 (38.2) | 0.74 (0.40-1.34), p=0.320 | 15 (34.1) | 1.44 (0.74-2.81), p=0.287 |
| Age | median | 30 (54.5) | 1.00 | 19 (43.2) | 1.00 |
|  | above median | 25 (45.5) | 0.59 (0.33-1.06), p=0.079 | 25 (56.8) | **0.51 (0.26-0.99), p=0.046** |
| Size of pCRC | below median | 27 (49.1) | 1.00 | 22 (50.0) | 1.00 |
|  | above median | 28 (50.9) | 0.90 (0.50-1.61), p=0.721 | 22 (50.0) | 1.38 (0.71-2.68), p=0.339 |
| Grade of pCRC | Low | 48 (88.9) | 1.00 | 40 (90.9) | 1.00 |
|  | High | 6 (11.1) | 0.66 (0.26-1.67), p=0.381 | 4 (9.1) | 0.39 (0.09-1.62), p=0.194 |
| Sideness | Right | 12 (21.8) | 1.00 | 8 (18.2) | 1.00 |
|  | Left | 43 (78.2) | 0.85 (0.42-1.70), p=0.642 | 36 (81.8) | 0.70 (0.31-1.62), p=0.410 |
| N grade | 0 | 13 (23.6) | 1.00 | 13 (29.5) | 1.00 |
|  | 1-2 | 42 (76.4) | 1.19 (0.61-2.30), p=0.613 | 31 (70.5) | 1.18 (0.59-2.34), p=0.645 |
| Median size of LM | below median | 31 (56.4) | 1.00 | 17 (38.6) | 1.00 |
|  | above median | 24 (43.6) | 0.83 (0.46-1.49), p=0.531 | 27 (61.4) | 1.10 (0.56-2.14), p=0.784 |
| Number of LM | below median | 25 (46.3) | 1.00 | 25 (58.1) | 1.00 |
|  | above median | 29 (53.7) | 1.26 (0.70-2.26), p=0.441 | 18 (41.9) | 1.55 (0.79-3.06), p=0.202 |
| Grade of LM | Low | 21 (39.6) | 1.00 | 18 (42.9) | 1.00 |
|  | High | 32 (60.4) | 0.84 (0.46-1.52), p=0.563 | 24 (57.1) | 1.32 (0.68-2.54), p=0.412 |
| Margin of LM | R0 | 40 (76.9) | 1.00 | 29 (69.0) | 1.00 |
|  | R1 | 12 (23.1) | 1.53 (0.77-3.04), p=0.222 | 13 (31.0) | 1.52 (0.74-3.10), p=0.254 |

Notes: Bold values indicate statistical significance at the p < 0.05 level. Median age: synchronous = 62, metachronous = 64; median size for primary tumor: synchronous = 4.3 cm, metachronous = 3.5 cm; median size of liver metastasis: synchronous = 2.0 cm, metachronous = 2.7 cm; median number of liver metastasis: synchronous = 2.0, metachronous = 2.1.

Abbreviations: TTR, time to recurrence; pCRC: primary colorectal cancer; LM: liver metastases; CRC: colorectal cancer; HR: hazard ratio; CI: confidence interval; OS: overall survival.

**Table S11**. Hazard ratios for DFS and TTR between above median vs below median Foxp3/CD4 ratio in OM of pCRC and IM/OM ratio of Foxp3+ T cell in LM of metachronous group* (multivariable analysis).

| Cell type and location along with confounders | Time to recurrence | | Disease-free survival | |
| --- | --- | --- | --- | --- |
|  | Group | HR (95% CI), p-value | Group | HR (95% CI), p-value |
| Foxp3/CD4 ratio in OM of pCRC | Above median | 2.02 (0.95-4.31), p=0.069 | Above median | 2.02 (0.95-4.31), p=0.069 |
| age | Above median | 0.64 (0.31-1.35), p=0.243 | Above median | 0.64 (0.31-1.35), p=0.243 |
|  | | | | |
| Foxp3/CD4 ratio in OM of pCRC | Above median | **2.50 (1.21-5.15), p=0.013** | Above median | **2.50 (1.21-5.15), p=0.013** |
| Chemotherapy regimen | FOLFOX vs other | 1.71 (0.79-3.71), p=0.175 | FOLFOX vs other | 1.71 (0.79-3.71), p=0.175 |
|  | | | | |
| IM/OM ratio of Foxp3+ T cell in LM | Above median | **0.45 (0.23-0.89), p=0.022** | Above median | **0.45 (0.23-0.89), p=0.022** |
| age | Above median | 0.61 (0.31-1.20), p=0.150 | Above median | 0.61 (0.31-1.20), p=0.150 |
|  | | | | |
| IM/OM ratio of Foxp3+ T cell in LM | Above median | **0.41 (0.21-0.80), p=0.009** | Above median | **0.41 (0.21-0.80), p=0.009** |
| Chemotherapy regimen | FOLFOX vs other | 1.29 (0.64-2.59), p=0.471 | FOLFOX vs other | - 1. (0.64-2.59), p=0.471 |

*There were no significant associations of clinical variables with outcomes in synchronous group.

Abbreviations: pCRC: primary colorectal cancer; LM: liver metastases; HR: hazard ratio; CI: confidence interval; IM: inner margin; OM: outer margin.

**Table S12.** Association between timing of chemotherapy and outcomes in CRC patients with synchronous and metachronous metastases

|  |  | synchronous | | metachronous | |
| --- | --- | --- | --- | --- | --- |
|  | Group | N | HR (95% CI), p-value | N | HR (95% CI), p-value |
| DFS | Before LS | 37 (67.3) | - | 11 (25.0) | - |
|  | After LS | 12 (21.8) | 0.88 (0.45-1.73, p=0.716) | 32 (72.7) | 0.58 (0.27-1.23, p=0.153) |
| TTR | Before LS | 37 (67.3) | - |  | - |
|  | After LS | 12 (21.8) | 0.91 (0.46-1.79, p=0.788) |  | 0.58 (0.27-1.23, p=0.153) |
| OS | Before LS | 37 (67.3) | - |  | - |
|  | After LS | 12 (21.8) | 0.61 (0.28-1.29, p=0.196) |  | 0.66 (0.28-1.56, p=0.347) |

DFS: disease-free survival; TTR: time to recurrence; OS: overall survival; LS: liver surgery

**Table S13.** Association between immune cells and clinical and pathological variables in CRC patients with synchronous and metachronous metastases

| Variables | Cell type and location | Synchronous | | | | Metachronous | | | |
| --- | --- | --- | --- | --- | --- | --- | --- | --- | --- |
|  |  | TC | IM | OM | PT | TC | IM | OM | PT |
| Male  Female | CD4pCRC  CD4 LM  CD4 pCRC  CD4 LM | 106 (1-758)*  50 (1-1724)  19 (0-218)  49 (0-1774) | 75 (1-505)  114 (0-2111)  38 (0-359)  76 (0-1931) | 150(1-1224)*  227(0-1878)  84(0-380)  170(0-1537) | 140(1-1354)*  188(6-1203)  70(0-412)  80(0-1053) | 50(0-1697)  91(0-1335)  76(0-532)  30(1-942) | 55(0-603)  205(0-1001)  30(0-987)  75(7-967) | 76(0-1202)  475(1-1301)  95(0-712)  250(10-1696) | 39(0-500)  210(5-1129)  120(0-1001)  406(2-954) |
| Male  Female | Foxp3 pCRC  Foxp3 LM  Foxp3 pCRC  Foxp3 LM | 99 (4-502)  27 (2-388)  108 (5-445)  18 (0-321) | 79 (15-542)  37 (3-479)  78 (21-312)  34 (7-377) | 108(11-813)  41(1-674)  100(27-301)  45(6-268) | 54(5-931)  13(1-187)  63(10-253)  9(2-60) | 131(16-590)  54(0-311)  194(12-1044)  25(0-269) | 111(6-469)  66(1-895)  164(6-1090)  35(5-284) | 134(9-781)  74(3-454)  126(9-1190)  54(6-556) | 45(0-287)  17(2-158)  45(8-524)  23(1-348) |
| Age  Above median  Below median | CD4pCRC  CD4 LM  CD4 pCRC  CD4 LM | 52 (0-435)  49 (0-850)  61 (0-758)  69 (0-1774) | 45 (0-390)  76 (0-1950)  71 (1-505)  114 (0-2111) | 129(0-1224)  200(90-847)  110(2-586)  246(0-1878) | 85(0-1354)  86(0-1053)  90(0-456)  173(0-1203) | 41(0-532)  37(1-942)  60(0-1697)  81(0-1335) | 28(0-987)  146(9-967)  80(0-603)  165(0-1001) | 76(0-712)  475(9-1696)  95(0-1202)  245(1-1301) | 42(1-1001)  368(2-1129)  120(0-505)  210(5-954) |
| Age  Above median  Below median | Foxp3 pCRC  Foxp3 LM  Foxp3 pCRC  Foxp3 LM | 100 (5-502)  22 (2-293)  95 (4-445)  29 (0-388) | 78 (21-542)  40 (3-297)  95 (15-411)  30 (4-479) | 110(27-813)  41(1-232)  98(11-301)  45(4-674) | 70(19-931)  13(1-187)  55(5-127)  13(2-143) | 106(19-506)  26(0-269)  201(12-1044)  50(0-311) | 82(6-568)  32(1-213)  230(6-1090)  55(2-895) | 99(21-455)  52(3-469)  180(9-1190)  67(4-556) | 38(12-174)  21(2-154)  55(0-524)  17(1-348) |
| Left-sided    Right-sided | CD4pCRC  CD4 LM  CD4 pCRC  CD4 LM | 60 (0-758)  50 (0-1774)  91 (3-435)  40 (5-187) | 60(0-505)  91(0-2111)  62(2-390)  80(10-324) | 110(0-550)  223(0-1878)  170(2-1224)  162(20-514) | 82(0-823)  165(0-1203)  141(0-1354)  118(16-562) | 52(0-1697)  54(0-1335)  39(0-532)  62(1-942) | 60(0-603)  148(0-1001)  34(0-987)  130(3-967) | 95(0-1202)  400(9-1301)  46(0-712)  238(1-1696) | 75(0-505)  300(9-954)  32(1-1001)  204(2-1129) |
| Left-sided  Right-sided | Foxp3 pCRC  Foxp3 LM  Foxp3 pCRC  Foxp3 LM | 94 (4-445)  18 (0-388)  128 (16-502)  35 (4-276) | 78(15-411)  30(3-479)  79(25-542)  54(5-297) | 112(11-415)  45(4-674)  82(27-813)  53(1-220) | 58(5-253)  13(1-143)  38(19-931)  13(2-187) | 139(12-1044)  52(0-311)  165(60-356)  23(3-206) | 109(6-1090)  51(1-895)  230(41-431)  44(4-213) | 122(9-1190)  66(3-556)  204(72-269)  65(4-179) | 42(0-524)  17(1-348)  64(30-110)  36(2-76) |
| pCRC size  Above median  Below median | CD4pCRC  CD4 LM  CD4 pCRC  CD4 LM | 52(0-280)  38(0-1724)  60(0-758)  69(0-1774) | 62(0-410)  133(0-2111)  60(1-505)  76(0-1931) | 142(0-586)  220(0-1878)  94(1-1224)  211(0-1537) | 83(0-499)  86(0-1203)  93(1-1354)  158(0-812) | 52(0-720)  54(0-1335)  50(0-1697)  65(4-942) | 31(0-484)  133(0-1001)  59(0-987)  152(7-967) | 110(0-1200)  259(1-1301)  85(0-1202)  288(9-1696) | 100(0-500)  213(2-920)  58(0-1001)  300(9-1129) |
| pCRC size  Above median  Below median | Foxp3 pCRC  Foxp3 LM  Foxp3 pCRC  Foxp3 LM | 127(4-445)  20(4-388)  93(5-502)  33(0-321) | 125(19-411)  34(5-322)  70(15-542)  44(3-479) | 103(22-415)  35(1-674)  112(11-813)  45(4-268) | 51(5-253)  13(2-187)  60(7-931)  12(1-143) | 147(16-590)  50(2-269)  136(12-1044)  41(0-311) | 130(6-469)  49(2-270)  92(6-1090)  50(1-895) | 172(9-781)  65(4-469)  100(9-1190)  66(3-556) | 61(0-287)  19(2-158)  38(8-524)  21(1-348) |
| pCRC grade 1  pCRC grade 2  pCRC grade 3 | CD4pCRC  CD4 LM  CD4 pCRC  CD4 LM CD3 CD4 pCRC  CD4 LM CD3 | 32(1-288)  28(1-512)  71(0-435)  69(0-1774)  13(0-758)  50(17-230) | 38(2-336)  73(1-1185)  63(0-410)  114(0-2111)  16(7-505)  52(24-319) | 121(4-439)  220(1-680)  142(0-1224)  173(0-1878)  44(1-400)  151(16-600) | 104(20-428)  122(6-720)  85(0-1354)  180(0-1203)  22(1-228)  31(18-401) | 50(0-720)  39(0-942)  56(0-532)  87(1-1335)  20(0-1697)  106(18-352) | 38(0-987)  93(0-967)  74(0-564)  150(7-1001)  28(0-603)  280(28-720) | 90(0-1200)  191(1-1696)  88(0-656)  475(40-901)  135(0-1202)  228(10-814) | 75(0-1001)  194(5-1129)  54(1-505)  300(2-954)  68(0-387)  301(181-421) |
| pCRC grade 1  pCRC grade 2  pCRC grade 3 | Foxp3 pCRC  Foxp3 LM  Foxp3 pCRC  Foxp3 LM Foxp3 pCRC  Foxp3 LM | 90(16-445)  15(2-139)  127(4-502)  27(0-388)  84(8-316)  26(6-36) | 70(34-136)  18(3-479)  111(15-542)  45(4-377)  134(35-180)  34(7-66) | 83(11-191)  27(1-135)  121(22-813)  47(4-674)  89(28-165)  47(10-109) | 29(7-111)  18(1-44)  70(5-931)  13(3-187)  66(10-99)  17(2-30) | 106(16-590)  32(0-206)  151(19-1044)  47(0-269)  91(12-500)  65(41-311) | 95(6-568)  44(1-213)  123(41-1090)  40(4-284)  84(15-461)  103(61-895) | 143(9-781)  65(3-179)  130(47-1190)  55(4-556)  64(9-159)  82(54-169) | 47(0-287)  21(2-76)  47(12-524)  16(1-348)  25(8-79)  39(31-40) |
| N stage 0  N stage 1  N stage 2 | CD4pCRC  CD4 LM  CD4 pCRC  CD4 LM  CD4 pCRC  CD4 LM | 13(3-140)  40(0-533)  61(0-330)  49(0-1724)  138(2-758)  69(1-1774) | 25(2-130)*  54(0-1636)  80(0-410)  91(0-2111)  70(1-505)  139(0-1950) | 87(1-394)  163(0-670)  150(0-564)  104(0-847)*  73(5-1224)  355(0-1878) | 83(0-823)  118(0-360)  105(0-499)  122(0-1053)  76(2-1354)  242(30-1203) | 73(0-720)  31(3-100)  21(0-293) *  93(0-544)  80(18-1697)  140(4-1335) | 49(0-564)  86(3-434)  28(0-194)*  148(0-720)  135(3-987)  215(13-1001) | 130(0-1200)  221(1-1301)  33(0-331)  370(9-900)  118(11-1202)  475(40-1696) | 150(0-500)  205(5-920)  28(0-285)  225(2-1129)  100(10-1001)  342(50-954) |
| N stage 0  N stage 1  N stage 2 | Foxp3 pCRC  Foxp3 LM  Foxp3 pCRC  Foxp3 LM Foxp3 pCRC  Foxp3 LM | 101(16-200)  24(2-90)  91(5-445)  22(0-388)  128(4-502)  25(2-321) | 57(21-260)  30(4-244)  111(21-411)  39(5-479)  135(15-542)  36(3-377) | 74(27-138)*  46(6-135)  128(11-301)  40(1-232)  113(22-813)  63(4-674) | 32(10-150)*  14(2-41)  81(7-253)  12(2-143)  56(5-931)  13(1-187) | 153(12-590)  38(0-147)  109(16-506)  33(0-269)  201(75-1044)  75(2-311) | 105(15-568)  47(5-237)  89(6-367)  51(1-242)  230(53-1090)  114(4-895) | 137(9-781)  63(7-556)  99(9-505)  57(3-469)  184(47-1190)  74(4-519) | 55(8-287)  22(1-158)  41(0-174)  19(2-154)  72(12-524)  21(2-348 |
| Number of LM  1  > 1 | CD4 pCRC  CD4 LM  CD4 pCRC  CD4 LM | 23 (0-435)  33 (0-1774)  61 (1-758)  69 (0-1724) | 40 (0-390)  55 (0-1950)  62 (2-505)  121 (0-2111) | 90 (0-1224)  173 (0-1093)  142 (1-586)  223 (0-1878) | 76 (0-1354)  113 (0-1053)  105 (1-823)  180 (0-1203) | 44 (0-532)  93 (0-942)  60 (0-1697)  30 (1-1335) | 30 (0-987)  170 (0-967)  80 (0-603)  100 (3-1001) | 110 (0-712)  505 (10-1696)  61 (0-1202)  178 (1-960)^0.024^ | 100 (0-1001)  368 (35-1129)  32 (1-500)  188 (2-848) |
| Number of LM  1  > 1 | Foxp3 pCRC  Foxp3 LM  Foxp3 pCRC  Foxp3 LM | 93 (4-502)  17 (0-321)  127 (5-445)  30 (4-388) | 78 (15-542)  30 (3-377)  76 (21-411)  44 (7-479) | 100 (22-813)  40 (1-268)  110 (11-301)  45 (6-674) | 56 (5-931)  12 (1-44)  57 (7-150)  18 (2-187) | 129 (12-1044)  54 (2-269)  196 (54-590)  23 (0-311) | 95 (6-1090)  87 (2-284)  132 (18-568)  36 (1-895) | 124 (9-1190)  86 (4-556)  182 (72-781)  46 (3-169)^0.032^ | 39 (0-524)  25 (2-348)  56 (13-287)  19 (1-63) |
| Size of LM  Below median  Above median | CD4 pCRC  CD4 LM  CD4 pCRC  CD4 LM | 61 (0-435)  70 (0-1774)  48 (1-758)  38 (0-509) | 60 (0-410)  146 (0-2111)  62 (2-505)  58 (0-873) | 139 (0-1224)  173 (0-1537)  117 (1-439)  241 (0-1878) | 83 (0-1354)  80 (0-812)  100 (0-412)  173 (0-1203) | 49 (0-1697)  31 (1-942)  55 (0-559)  58 (0-1335) | 30 (0-987)  121 (3-967)  74 (0-410)  175 (0-1001) | 45 (0-1202)  230 (1-1696)  138 (0-420)  510 (10-1301) | 35 (1-1001)  225 (2-1129)  105 (0-505)  355 (35-954) |
| Size of LM  Below median  Above median | Foxp3 pCRC  Foxp3 LM  Foxp3 pCRC  Foxp3 LM | 113 (6-502)  27 (0-388)  98 (4-448)  18 (2-293) | 133 (15-542)  40 (3-479)  70 (19-311)^0.054^  34 (4-256) | 125 (11-813)  27 (4-268)  100 (22-301)  60 (1-674) | 68 (7-931)  10 (1-143)  38 (5-253)  21 (2-187) | 131 (43-500)  50 (0-311)  184 (12-1044)  41 (2-269) | 90 (18-470)  44 (1-895)  130 (6-1090)  51 (2-284) | 100 (24-781)  65 (3-556)  172 (9-1190)  66 (4-519) | 44 (13-287)  22 (1-158)  49 (0-524)  17 (2-348) |
| Grade LM 1  Grade LM 2 | CD4 pCRC  CD4 LM  CD4 pCRC  CD4 LM | 80 (0-288)  49 (6-512)  48 (0-758)  50 (0-1774) | 62 (2-280)  110 (9-1185)  48 (0-505)  65 (0-2111) | 149 (1-395)  226 (16-1878)  90 (0-1224)  173 (0-1537) | 105 (0-428)  136 (16-1203)  82 (0-1354)  126 (0-812) | 49 (0-720)  35 (0-1335)  56 (0-1697)  87 (1-544) | 50 (0-987)  86 (0-1001)  49 (0-603)  154 (7-921) | 61 (0-1200)  160 (1-1696)  114 (0-1202)  500 (10-960)^0.042^ | 35 (0-1001)  183 (2-920)  105 (0-505)  355 (25-1129) |
| Grade LM 1  Grade LM 2 | Foxp3 pCRC  Foxp3 LM  Foxp3 pCRC  Foxp3 LM | 113 (5-445)  20 (5-193)  98 (4-502)  29 (0-388) | 78 (19-311)  37 (10-479)  78 (15-542)  31 (3-377) | 83 (11-301)  39 (11-674)  117 (22-813)  45 (1-268) | 47 (7-253)  11 (2-187)  67 (5-931)  14 (1-143) | 139 (16-590)  26 (0-211)  144 (12-1044)  51 (0-311) | 83 (6-469)  47 (1-270)  175 (15-1090)  55 (4-895) | 92 (9-781)  55 (3-179)  138 (9-1190)  71 (4-556) | 45 (0-287)  15 (2-63)  47 (8-524)  26 (1-348) |
| LM Margin R0  LM Margin R1 | CD4 pCRC  CD4 LM  CD4 pCRC  CD4 LM | 54 (0-758)  49 (0-1774)  113 (0-288)  132 (1-1724) | 63 (0-505)  73 (0-1950)  57 (1-410)  232 (0-2111) | 117 (0-1224)  231 (0-1537)  121 (5-345)  68 (0-1878) | 104 (0-1354)  195 (0-1053)  63 (11-428)  50 (6-1203) | 44 (0-720)  84 (0-1335)  60 (0-1697)  39 (1-260) | 30 (0-564)  121 (0-1001)  49 (0-603)  150 (3-519) | 90 (0-1200)  299 (9-900)  84 (0-1202)  186 (1-1301) | 75 (0-500)  300 (9-1129)  50 (0-505)  183 (2-954) |
| LM Margin R0  LM Margin R1 | Foxp3 pCRC  Foxp3 LM  Foxp3 pCRC  Foxp3 LM | 110 (4-502)  231 (0-321)  90 (8-445)  26 (2-388) | 78 (19-542)  34 (3-377)  70 (15-411)  35 (4-479) | 106 (22-813)  47 (1-268)  76 (11-269)  30 (4-674) | 57 (5-931)  13 (1-65)  51 (7-111)  6 (2-187) | 129 (16-506)  43 (0-269)  165 (12-1044)  41 (0-311) | 95 (6-568)  45 (1-270)  123 (15-1090)  47 (4-895) | 124 (9-781)  66 (3-556)  55 (4-519)  143 (9-1190) | 42 (0-287)  20 (2-158)  39 (8-524)  18 (1-348) |

Notes: ^&^: median (min-max), *: P<0.05

Median age: synchronous = 62, metachronous = 64; median size for primary tumor: synchronous = 4.3 cm, metachronous = 3.5 cm; median size of liver metastasis: synchronous = 2.0 cm, metachronous = 2.7 cm.

Abbreviations: pCRC: primary colorectal cancer; LM: liver metastases; CRC: colorectal cancer.

**Table S14.** Association between immune cells and chemotherapy before and after liver resection in CRC patients with synchronous and metachronous metastases

| Variables | Cell type and location | Synchronous group (37 patients before and 12 after) | | | | Metachronous group (11 patients before and 32 after) | | | |
| --- | --- | --- | --- | --- | --- | --- | --- | --- | --- |
|  |  | TC | IM | OM | PT | TC | IM | OM | PT |
| CHT before liver surgery  CHT after liver surgery | CD4pCRC  CD4 LM  CD4 pCRC  CD4 LM | 33 (0-758)*  50 (0-1774)  73 (8-330)  70 (0-367) | 45 (0-505)  104 (0-2111)  63 (2-350)  80 (0-873) | 117 (0-1224)  185 (0-1878)  117 (1-490)  300 (0-680) | 80 (0-1354)  99 (0-1203)  122 (0-300)  198 (0-720) | 60 (0-1697)  66 (4-1335)  50 (0-720)  43 (0-942) | 80 (0-603)  98 (7-1001)  50 (0-987)  165 (0-967) | 90 (0-1202)  140 (10-475)  110 (0-1200)  500 (1-1696)^0.0087^ | 66 (0-387)  135 (25-520)  100 (0-1001)  240 (2-1129) |
| CHT before liver surgery  CHT after liver surgery | Foxp3 pCRC  Foxp3 LM  Foxp3 pCRC  Foxp3 LM | 98 (4-502)  29 (0-388)  109 (5-240)  39 (4-293) | 78 (15-542)  34 (3-479)  94 (19-260)  58 (7-256) | 106 (11-813)  40 (1-674)  75 (26-212)  92 (10-231) | 57 (5-931)  13 (1-138)  31 (19-118)  33 (5-187) | 187 (12-590)  26 (0-311)  138 (16-1044)  47 (0-206) | 118 (6-461)  32 (4-895)  102 (6-1090)  59 (1-284) | 104 (9-409)  37 (4-169)  137 (9-1190)  74 (3-556)^0.049^ | 38 (8-139)  12 (1-47)  50 (0-524)  21 (2-348) |
| CHT before liver surgery  CHT after liver surgery | Foxp3/CD4 pCRC Foxp3/CD4 LM  Foxp3/CD4 pCRC Foxp3/CD4 LM | 2.7 (0.2-690.0)  0.5 (0-170.0)  1.4 (0-22.4)  0.6 (0.2-40.0) | 2.2 (0.2-1110.0)  0.3 (0-220.0)  0.9 (0-25.8)  0.7 (0.2-70.0) | 1.1 (0-830.0)  0.9 (0.2-10.5)  0.8 (0-100.0)  0.7 (0.3-1.5) | 0.7 (0-290.0)  0.1 (0-90.0)  0.5 (0-1160.0)  0.2 (0-80.0) | 2.8 (0.3-115.5)  0.2 (0-3.3)  2.3 (0-1060.0)  0.7 (0-40.0) | 1.5 (0.4-149.4)  0.3 (0-2.2)  2.1 (0.3-60.0)  0.5 (0.1-20.0) | 1.4 (0-85.9)  0.2 (0-5.4)  1.4 (0.3-274.0)  0.2 (0-63.0) | 0.6 (0-79.4)  0 (0-0.2)  0.7 (0.1-64.0)  0.1 (0-11.4) |

*Median (min-max) cell densities

Abbreviations: CRC: colorectal cancer; pCRC: primary colorectal cancer; LM: liver metastases; TC: tumor center; IM: inner margin; OM: outer margin; PT: peritumor zone; CHT: chemotherapy

**Table S15.** Association between immune cells and FOLFOX-based chemotherapy versus other in CRC patients with synchronous and metachronous metastases

| Chemotherapy  regimen | Cell type and location | Synchronous (31 patients FOLFOX and 18 other) | | | | Metachronous (16 patients FOLFOX and 23 other) | | | |
| --- | --- | --- | --- | --- | --- | --- | --- | --- | --- |
|  |  | TC | IM | OM | PT | TC | IM | OM | PT |
| FOLFOX  Other | Foxp3/CD4 pCRC Foxp3/CD4 LM  Foxp3/CD4 pCRC Foxp3/CD4 LM | 2.1 (0-53.0)*  0.7 (0-70.0)  3.0 (0.4-1110)  0.4 (0-170.0) | 1.6 (0-25.8)  0.5 (0-70.0)  2.5 (0.5-1110.0)  0.3 (0.1-220.0) | 0.9 (0-100.0)  0.8 (0.3-10.5)  1.1 (0.4-830.0)  0.9 (0.2-2.6) | 0.6 (0-1160.0)  0.1 (0-80.0)  0.8 (0.1-290.0)  0.2 (0-90.0) | 2.7 (0.3-13.6)  0.4 (0-13.0)  2.4 (0-1060.0)  0.5 (0-40.0) | 2.3 (0.4-35.7)  0.4 (0-1.9)  1.5 (0.3-60.0)  0.4 (0.1-20.0) | 2.2 (0.1-10.3)  0.2 (0-1.7)  1.4 (0.2-274.0)  0.2 (0-63.0) | 0.9 (0.1-4.1)  0.1 (0-10.5)  0.7 (0.1-64.0)  0.1 (0-11.4) |

* Median (min-max) ratio of cell densities

Abbreviations: pCRC: primary colorectal cancer; LM: liver metastases; TC: tumor center; IM: inner margin; OM: outer margin; PT: peritumor zone
